# Biochar Application Improves Soil Aggregate Stability and Aggregate-Associated Carbon Fractions Through Microbial Community Regulation in *Eucalyptus* Plantations—A Seven-Year Field Experiment

**DOI:** 10.3390/microorganisms14081847

**Published:** 2026-08-20

**Authors:** Jialin Liao, Yuyi Shen, Denan Zhang, Yingjie Sun, Qiumei Teng, Guangping Xu, Yunhuang Luo, Kechao Huang, Hao Shi, Zhiwen Tan, Junzhi Chu, Yu Cao

**Affiliations:** 1 Guangxi Key Laboratory of Plant Conservation and Restoration Ecology in Karst Terrain, Guangxi Institute of Botany, Guangxi Zhuang Autonomous Region and Chinese Academy of Sciences, Guilin 541000, China; liaojialin2026@163.com (J.L.); denanzhang@126.com (D.Z.); syj@gxib.cn (Y.S.); tqm1907@163.com (Q.T.); kchuanggxib@163.com (K.H.); shihao@gxib.cn (H.S.); 2 College of Life Sciences, Guangxi Normal University, Guilin 541000, China; 13246852164@163.com; 3 Guangxi Key Laboratory of Functional Phytochemicals and Sustainable Utilization, Guangxi Institute of Botany, Guangxi Zhuang Autonomous Region and Chinese Academy of Sciences, Guilin 541000, China; 4 Guangxi Guilin Urban Ecosystem Observation and Research Station, Guilin 541000, China; 5 College of Environmental Science and Engineering, Guilin University of Technology, Guilin 541000, China; 2120240612@glut.edu.cn (Y.L.); 18483190321@163.com (Y.C.); 6 The State Key Laboratory of Subtropical Silviculture, Zhejiang A&F University, Hangzhou 311300, China; 17636386112@163.com

**Keywords:** biochar, plantation, soil aggregates, bacteria, fungi, soil carbon fractions

## Abstract

Biochar has been used to improve soils and promote sustainable agricultural development. The effects of applying different doses of biochar on soil aggregate structure and stability, aggregate-associated microbial communities, aggregate carbon fractions, and underlying mechanisms in planted forest soil ecosystems remain unclear. This study aimed to explore the effects of biochar amendment (7 years) on carbon stabilization in plantation soils. The effects of biochar application (0%, 0.5%, 1.0%, 2%, 4%, and 6%) on water-stable aggregate distribution, stability indices such as mean weight diameter (MWD), geometric mean diameter (GMD), and fractal dimension (*D*), aggregate-associated microbial communities (fungal and bacterial phospholipid fatty acids (PLFAs)), and carbon fractions such as soil organic carbon (SOC), easily oxidized organic carbon (EOC), dissolved organic carbon (DOC), particulate organic carbon (POC), microbial biomass carbon (MBC), recalcitrant organic carbon (ROC) and black carbon (BC) were investigated based on a seven-year in situ field experiment in a *Eucalyptus* plantation in northern Guangxi. The results showed that after 7 years, biochar application significantly increased the proportion of macroaggregates (≥0.25 mm). The MWD and GMD increased significantly with increasing biochar application rates, whereas D decreased significantly, indicating enhanced soil structural stability. Biochar significantly increased the abundance of fungi and bacteria across all aggregate size classes and changed the microbial community structure towards conditions that promoted increased carbon stabilization. Additionally, biochar application significantly increased both recalcitrant (ROC and BC) and labile carbon (EOC, POC, DOC, and MBC) in all aggregate fractions, with the largest increments in macroaggregates. Based on correlation analysis and structural equation modeling (SEM), we speculated that biochar might enhance the physical protection and chemical sequestration of organic carbon by optimizing the physical structure of the aggregates and synergizing with the microbial carbon pump. The 7-year application of biochar significantly enhanced the SOC content in plantation soils, primarily by increasing recalcitrant organic carbon, demonstrating that biochar application constitutes a viable approach to augmenting persistent soil carbon stabilization in plantation ecosystems. The 4% and 6% treatments produced the largest responses for most of the measured indicators, underscoring the importance of biochar application.

## 1. Introduction

Soil constitutes the dominant organic carbon sink within land-based ecosystems, containing more carbon than the atmosphere and vegetation pools combined. Consequently, it is indispensable for modulating the global carbon cycle and alleviating climate change [1,2,3]. As fundamental units of soil structure, aggregates serve as primary sites for microbial activity and organic carbon sequestration and regulate microbial processes and the distribution of soil organic carbon (SOC) by influencing aggregate size distribution and stability [4,5,6]. Approximately 90% of organic carbon in surface soil is stored within aggregates, where it is stabilized primarily through physical protection and organo-mineral interactions [7,8,9]. Therefore, focusing on soil aggregates and their associated SOC content is of considerable importance for understanding total SOC sequestration. However, anthropogenic activities such as irrational fertilization and intensive forestry management practices, including continuous planting and shortened rotation periods, can severely disrupt the native hierarchical structure of the soil, accelerating aggregate breakdown. This structural degradation directly reduces the protective function of soil aggregates, thereby exacerbating soil carbon [10,11,12,13]. Currently, various traditional soil amelioration measures—including chemical fertilization, liming, and organic manure application—each have inherent limitations. Although these approaches can improve certain soil properties to some extent, they are unable to achieve long-term carbon stabilization [14,15]. Therefore, developing efficient and sustainable soil amendment strategies to enhance aggregate stability and increase soil carbon sequestration capacity is urgently required [16,17].

Biochar is widely considered an effective management strategy for improving soil quality, augmenting soil fertility, and increasing long-term carbon sequestration capacity owing to its high carbon content, substantial specific surface area, and porous architecture [18,19,20]. These physicochemical properties are largely governed by feedstock type and pyrolysis conditions, which in turn determine its performance as a soil amendment [21,22]. Specifically, Biochar acts as a cementing agent that facilitates the transformation of microaggregates into macroaggregates owing to its porous nature, high cation-exchange capacity, and cation-bridging effects. This process enhances the degree of soil aggregation and structural stability, while simultaneously providing carbon sources and habitats for soil microorganisms, which synergistically reinforce the aggregate structure [16,23,24,25]. The application of biochar notably increases soil aggregates, their stability (mean weight diameter [MWD]), and SOC content [1,16,26,27], with sustained effects observed even after long-term applications (>3 years) [1,27,28,29]. In addition to its role in aggregation, recent conceptual advances have further suggested that biochar may enhance carbon pool stability by acting as a “bridge” between the microbial carbon pump and the mineral carbon pump, facilitating the formation of microaggregates and stable organo-mineral complexes [30,31]. Despite these promising findings, current biochar studies have primarily focused on croplands or short-term trials [4,22,23,24,25,26,27,28,29,30,31,32,33,34,35,36,37], whereas long-term in situ experiments in plantation soils have rarely been reported. A short-term in situ study demonstrated that, in understory environments, the continuous application of pure biochar for two years conversely reduced the proportion of larger aggregates and aggregate stability [35]. This suggests that short-term observations may not capture the full trajectory of biochar effects, and that medium- to long-term field trials are essential to reveal the true potential of biochar in stabilizing SOC and its fractions within aggregates in plantation ecosystems [21,31,38]. SOC fractions include labile carbon pools such as easily oxidized organic carbon (EOC), particulate organic carbon (POC), microbial biomass carbon (MBC), and dissolved organic carbon (DOC), and stable carbon pools such as recalcitrant organic carbon (ROC). These fractions do not respond uniformly to external disturbances [1,39,40]. Additionally, existing studies frequently focus only on specific fractions and do not analyze different components, such as EOC, POC, MBC, DOC, and ROC, at the aggregate scale [37,41,42]. The role of black carbon (BC), a highly aromatic component resistant to microbial degradation, in aggregate carbon sequestration mechanisms has rarely been reported in long-term woodland biochar application experiments [36,43]. In situ observational evidence regarding the influence of biochar on the distribution of different aggregate size classes and the allocation of their internal carbon fractions over long timescales remains lacking.

Soil aggregates are crucial sites for microbial activity and nutrient cycling [44], harboring distinct microbial communities that occupy specific ecological niches within different aggregate size classes [39]. Soil microorganisms, particularly fungi and bacteria, are key biological drivers of SOC turnover and aggregate formation [45], which are vital in long-term SOC sequestration [39,46]. Biochar application alters soil aggregate composition and stability, which may indirectly influence aggregate-associated microbial activities and interactions, thereby affecting the turnover and stabilization of SOC [5]. Therefore, investigating the responses of microbial communities within different aggregate size classes to biochar application is essential for a comprehensive understanding of the soil carbon sequestration capacity of forest ecosystems [47].

*Eucalyptus urophylla* × *E. grandis* is one of the most widely planted fast-growing tree species globally, owing to its rapid growth, high adaptability, and substantial economic returns. This is particularly evident in Guangxi, the hub of China’s *Eucalyptus* plantation industry, which accounts for over half of the total national *Eucalyptus* plantation area. However, the contradiction between the substantial demand for soil nutrients and the decline in soil fertility during tree growth has become increasingly prominent. To maintain and improve the sustainable productivity of fast-growing plantations, biochar application may be a sustainable method with considerable potential for coordinating the nutrient supply and soil health [1]. Simultaneous investigations of the responses of aggregate stability, aggregate organic carbon fractions, and aggregate-associated microorganisms to biochar application in plantation soils remain scarce. Consequently, based on an in situ field experiment in a *Eucalyptus* plantation in northern Guangxi, this study aimed to evaluate the impacts of a single biochar amendment after seven years on soil aggregate stability, aggregate-associated microbial community structure, and organic carbon fractions. We hypothesize that biochar promotes the accumulation of recalcitrant organic carbon by optimizing soil aggregate structure and modulating aggregate-associated microbial communities, thereby enhancing the stability of the soil carbon pool. The specific study objectives were as follows: (1) to evaluate the influence of biochar application on soil aggregate size distribution and stability; (2) to quantify the accumulation and distribution characteristics of SOC fractions within aggregate size classes; and (3) to elucidate the relationships among aggregate stability, aggregate-associated microorganisms, and carbon fractions following seven years of biochar application and how these interactions influence soil carbon stabilization capacity.

## 2. Materials and Methods

### 2.1. Experimental Design and Soil Sampling

The study area was situated in the state-owned Huangmian Forest Farm (24°37′25″–24°52′11″ N, 109°43′46″–109°58′18″ E) at the junction of Luzhai County (Liuzhou City) and Yongfu County (Guilin City) in Guangxi. The region exhibits a mid-subtropical climatic zone, characterized by a humid climate, distinct seasons, and concurrent rainfall and heat. Precipitation is mainly concentrated from April to August, with a mean annual precipitation of 1750–2000 mm. The mean annual temperature is 19 °C, indicating optimal thermal conditions, and an annual average evaporation is 1426–1650 mm. The Huangmian Forest Farm is characterized by mountainous and hilly terrain with notable undulations and steep slopes. The soil in the *Eucalyptus* plantation is primarily red soil and mountain yellow-red soil developed from parent materials such as sandstone and sandy shale [1].

This study was an extension of an in situ biochar application experiment in a *Eucalyptus* plantation established in March 2017 [1,16]. The biochar utilized in the experiment was fabricated from the waste branches of harvested *Eucalyptus* trees from the experimental base. It was prepared by pyrolysis and carbonization at a high temperature (500 °C) under anaerobic conditions for 2 h by Jining Dehanqi Mechanical Engineering Technology Co., Ltd. (Jining, China). The prepared biochar was then milled and screened to a 2 mm sieve to ensure homogeneous particle dimensions. The basic physicochemical properties of the biochar were as follows: specific surface area 43.21 m^2^/g, electrical conductivity 692.82 μS/cm, cation exchange capacity 51.48 cmol/kg, ash content 41.05%, pH 9.52, carbon content 55.86%, hydrogen content 3.13%, oxygen content 23.70%, nitrogen content 1.36%, phosphorus content 5.46 mg/g, and potassium content 32.48 mg/g. The experimental plots were selected from representative *Eucalyptus* plantations with similar parent materials, comparable altitudes, relatively gentle topography, and moderate slopes. The stand density was 1250 trees/ha, with a planting pattern of narrow spacing (2 m × 3 m) and wide row spacing of 5 m. The initial physicochemical properties of the experimental soil were: pH 5.39, soil water content 20.28%, SOC content 8.44 g/kg, total nitrogen (TN) 1.09 g/kg, total phosphorus (TP) 0.38 g/kg, total potassium (TK) 6.79 g/kg, available nitrogen (AN) 72.78 mg/kg, available phosphorus (AP) 1.61 mg/kg, and available potassium (AK) 40.22 mg/kg. Biochar was applied following the protocols outlined by Guo et al. [48] and Zhou et al. [49], with application rates controlled based on the mass percentage of biochar to dry soil weight. Six treatments were established: 0% (CK), 0.5% (T1), 1.0% (T2), 2% (T3), 4% (T4), and 6% (T5). Each treatment was replicated thrice. To ensure data reliability, a randomized complete block design (RCBD) was employed, consisting of 18 plots in total. Each plot measured 8 × 8 m and was separated by a 1 m buffer zone to prevent cross-contamination between plots. The biochar was applied using a complete mixing method. Specifically, biochar was thoroughly homogenized with the tilled soil at the predetermined experimental ratios, and the mixed soil was backfilled in situ and gently compacted to restore the pre-tilling soil state as much as possible. The control plots were tilled similarly without the addition of biochar. No additional fertilizer was applied during the experiment, and biochar was applied only once to observe its long-term effects.

Soil samples were collected from the *Eucalyptus* plantation in northern Guangxi in March 2024. Five representative sampling sites were randomly selected from each plot, using an “S-shaped” sampling pattern. The soil was collected from three layers: 0–10 cm (surface layer), 10–20 cm (middle layer), and 20–30 cm (subsurface layer), using a professional soil auger. Five subsamples from the same layer within each plot were mixed to form a composite sample. Visible litter and plant residues were removed from the composite samples, which were subsequently placed in sterile sealed bags and immediately stored in ice pack containers. Soil samples were collected from three replicate plots per treatment (biological replicates, n = 3). After sampling, the samples were transported to the laboratory and stored at 4 °C [50] for the analysis of soil aggregates, microbial phospholipid fatty acids (PLFAs), and soil carbon fractions. For each sample, laboratory analyses were performed in triplicate (analytical replicates, n = 3), and the mean values were used for statistical analysis.

### 2.2. Soil Physicochemical Analysis

#### 2.2.1. Soil Aggregate Size Distribution and Stability Indices

The field-collected soil samples were passed through an 8 mm sieve and placed on the top sieve (2 mm) of a nested set consisting of 2.000, 0.250, and 0.053 mm meshes. The samples were fully submerged in water and soaked for 15 min. Subsequently, wet sieving was performed using an aggregate analyzer (TPF-100) at an oscillation speed of 30 rpm for 30 min [51]. After sieving, the aggregates retained on each sieve were flushed into beakers to separate four classes of water-stable aggregates: large macroaggregates (>2 mm), small macroaggregates (2–0.25 mm), microaggregates (0.25–0.053 mm), and silt-clay aggregates (<0.053 mm). This process was repeated to obtain sufficient aggregate samples for the analyses of size distribution, stability indices, PLFAs, and soil carbon fractions. The collected aggregates of each particle size were oven-dried at 60 °C for the analysis of size distribution and carbon fractions. For MBC and DOC measurements, separate fresh subsamples of each aggregate fraction were retained prior to oven-drying. These subsamples were stored at 4 °C in sealed containers and processed within 48 h of sampling. The soil samples retained on each sieve were weighed to calculate mean weight diameter (MWD), geometric mean diameter (GMD), proportion of aggregates >0.25 mm (R0.25), and fractal dimension (*D*) to characterize the soil aggregate stability [35,52].

#### 2.2.2. Analysis of Aggregate-Associated Microbial Communities (PLFAs)

Fresh soil aggregate samples (4.0 g) from each size fraction were weighed for the extraction of total lipids using chloroform-methanol-citrate buffer (1:2:0.8, *v*/*v*/*v*) following the Bligh–Dyer method [53]. Phospholipid fatty acids (PLFAs) were separated from the total lipids via silica gel column chromatography. After methylation, the PLFA contents of fungi, bacteria, Gram-positive bacteria, and Gram-negative bacteria were determined using an Agilent 6890 Gas Chromatograph (Hewlett-Packard 6890; Agilent Technologies, Santa Clara, CA, USA), with esterified C19:0 as the internal standard. Microbial taxa were identified using the Sherlock Microbial Identification System (MIS) 4.5 (MIDI, Inc., Newark, DE, USA), and fungi, bacteria, Gram-positive bacteria, and Gram-negative bacteria were classified based on specific PLFA biomarkers [54]. Specifically, bacterial PLFAs comprised i15:0, a15:0, i16:0, i17:0, a17:0, cy17:0, 16:1ω7c, 17:1ω8, 17:1ω9c, 18:0, 18:1ω5, 18:1ω7c, and cy19:0. Gram-positive bacteria were identified by i15:0, a15:0, i16:0, i17:0, and a17:0, whereas Gram-negative bacteria were characterized by cy17:0, 16:1ω7c, 17:1ω9c, 18:1ω5, and 18:1ω7c. Fungal PLFAs were represented by 16:3ω6c, 18:2ω6, 9c, and 18:1ω9c.

#### 2.2.3. Analysis of Aggregate-Associated Carbon Fractions

Sieved soil aggregates from each size class were used to determine carbon fractions. The contribution rates of carbon fractions in aggregates of each size fraction to total SOC was calculated based on a previously described method [55]. SOC was determined using a Total Organic Carbon Analyzer (Shimadzu 5000A, Kyoto, Japan). EOC, DOC, POC, and MBC were analyzed by the potassium permanganate oxidation-colorimetric method [56], the 0.5 mol·L^−1^ K_2_SO_4_ extraction method [57], the sodium hexametaphosphate dispersion method [58], and the chloroform fumigation-extraction method [59], respectively. Detailed experimental procedures for these parameters have been previously described [60]. The ROC was determined using the 6 N HCl acid hydrolysis method [61]. BC determination was based on a previously described method [62] with appropriate modifications. Briefly, 3.0 g of sieved soil sample was weighed, and 15 mL of 3 mol·L^−1^ HCl was added to remove carbonates. After reacting for 24 h, 15 mL of a solution containing 10 mol·L^−1^ HF and 1 mol·L^−1^ HCl was added to remove silicates. After another 24 h, 15 mL of 10 mol·L^−1^ HCl was added to remove potential CaF_2_ precipitates. Following a further 24 h reaction, 15 mL of a solution containing 0.1 mol·L^−1^ K_2_Cr_2_O_7_ and 2 mol·L^−1^ H_2_SO_4_ was added to oxidize organic carbon at 55 ± 1 °C for 65 h. The remaining residue, regarded as the BC sample, was centrifuged, dried, and analyzed for BC content using an Elemental Analyzer–Isotope Ratio Mass Spectrometer (FLASH EA–DELTA V; Thermo Fisher Scientific, Bremen, Germany). To calculate the carbon contribution rate, first multiply the proportion of each particle size class by its carbon content, then compute the percentage of this value relative to the total carbon content of the untreated raw soil (full soil).

### 2.3. Statistical Analysis

Data were subjected to Analysis of Variance (ANOVA) and Least Significant Difference (LSD) tests using SPSS V26.0 (IBM, Chicago, IL, USA) to analyze the differences in the effects of biochar application at varying rates on the particle size distribution and stability of soil aggregates, fungal and bacterial PLFAs, and carbon fractions of soil aggregates. All data met the assumptions of normality (*p* > 0.05) and homogeneity of variance (*p* > 0.05) before ANOVA was performed. Origin 2021 was used to generate visualizations, including bar charts, Pearson correlation heat maps, principal component analysis (PCA) plots, and linear regression analyses. Additionally, partial least squares structural equation modeling (PLS-SEM) was performed using SmartPLS 4 to investigate the associations between aggregate stability, aggregate-associated fungal and bacterial abundance, and organic carbon fractions under biochar amendment. Additionally, partial least squares structural equation modeling (PLS-SEM) was performed using SmartPLS 4 to investigate the associations between aggregate stability, aggregate-associated fungal and bacterial abundance, and organic carbon fractions under biochar amendment. The model was constructed based on the following hypothesized pathways: biochar influences SOC indirectly through altering aggregate stability (R_0.25_), microbial abundance (bacterial and fungal PLFAs), and labile/recalcitrant carbon fractions. Two latent variables were constructed using the following observed indicators: labile carbon pool: POC, EOC, DOC, and MBC; recalcitrant carbon pool: ROC and BC. Model fit was evaluated using the standardized root mean square residual (SRMR) and the goodness of fit (GOF). Internal consistency was assessed using Cronbach’s α and composite reliability. Path significance was determined using bootstrapping with 5000 resamples and 95% bias-corrected confidence intervals. All indicators were standardized prior to analysis.

## 3. Results

### 3.1. Responses of Soil Water-Stable Aggregation Composition and Stability to Graded Biochar Amendment Levels

**Figure 1:**
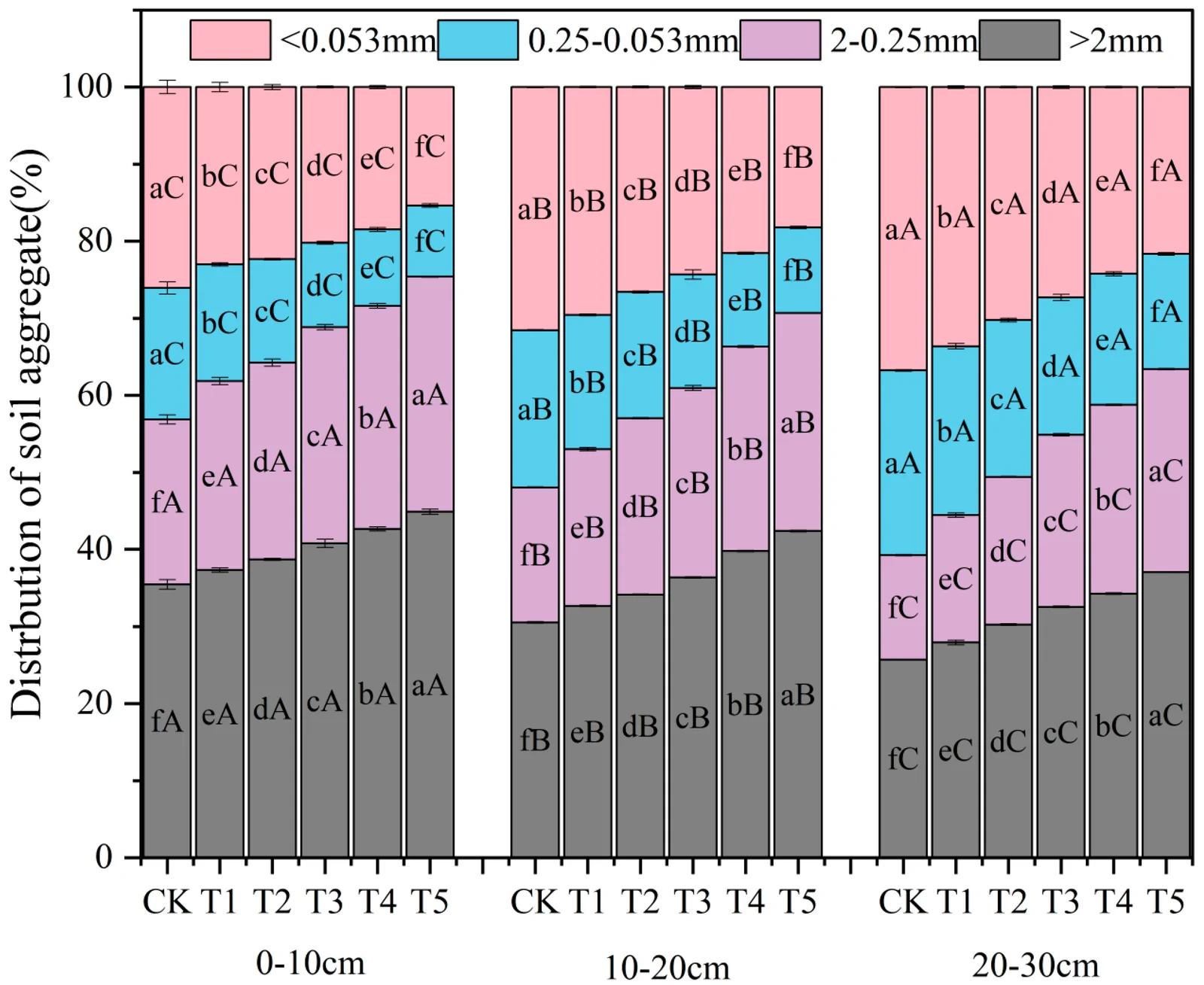
Effects of different biochar amendment levels on soil aggregate composition. Values are mean ± SD. The six treatments correspond to biochar application rates of CK (0%), T1 (0.5%), T2 (1.0%), T3 (2%), T4 (4%), and T5 (6%). Different lowercase letters indicate significant differences among treatments within the same soil layer (*p* < 0.05), and different uppercase letters indicate significant differences among soil layers within the same treatment (*p* < 0.05). The same as below.

Biochar application significantly affected the composition of water-stable soil aggregates. As illustrated in Figure 1, within the same soil layer, the macroaggregate proportion (≥0.25 mm) exhibited a significant increasing trend with increasing biochar amendment levels, whereas microaggregate proportion (<0.25 mm) showed a significant decreasing trend. Under the same treatment, the macroaggregate proportion decreased significantly with increasing soil depth, whereas the microaggregate content increased significantly. Specifically, the macroaggregate proportion ranged from 56.86% to 75.40% in the 0–10 cm layer, from 48.04% to 70.69% in the 10–20 cm layer, and from 39.26% to 63.40% in the 20–30 cm layer, while the microaggregate proportion ranged from 24.60% to 43.14%, 29.31% to 51.96%, and 36.60% to 60.74%, respectively. Biochar significantly enhanced soil macroaggregate proportion, concomitantly decreasing microaggregate proportion.

**Figure 2:**
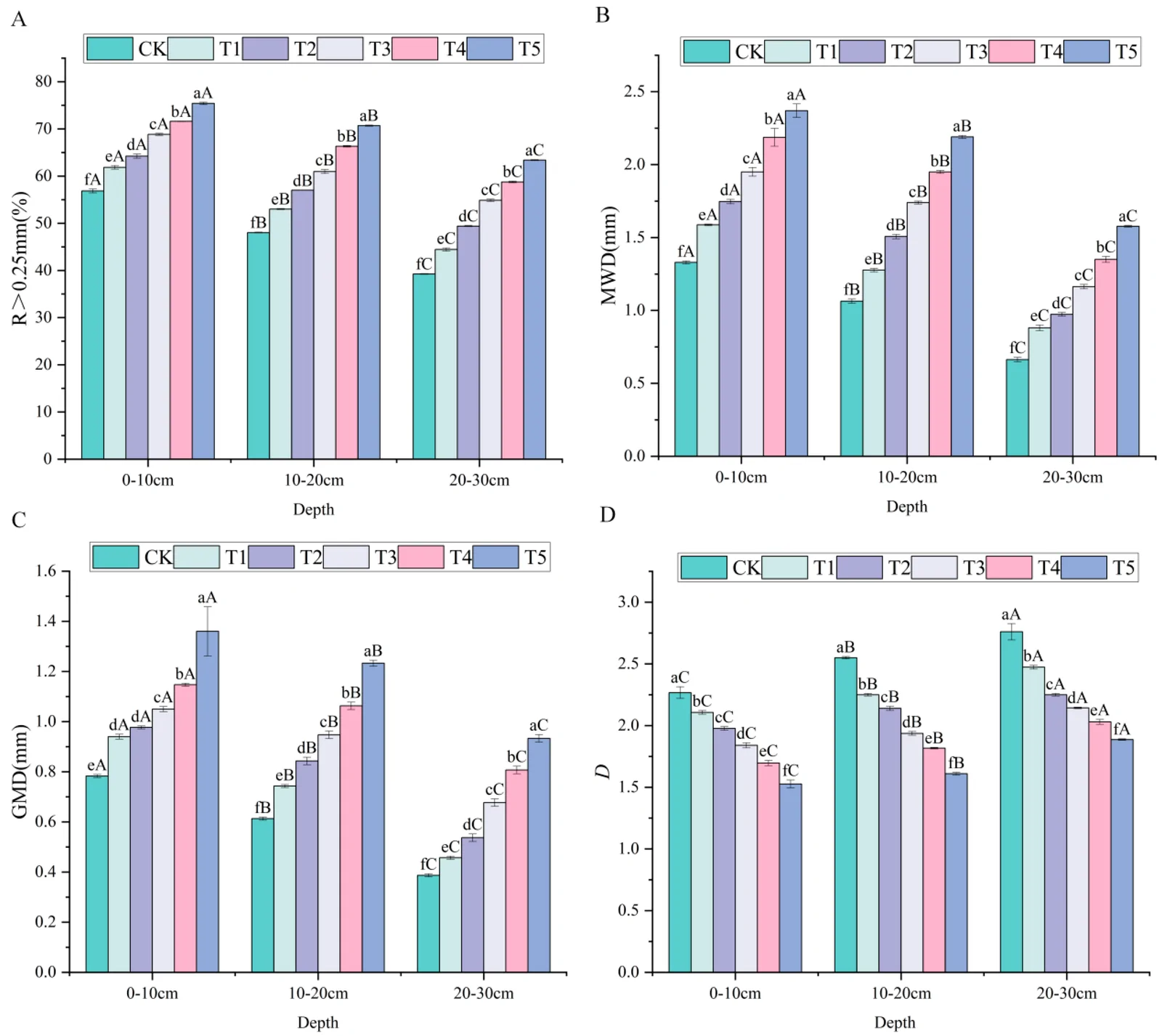
Effects of different biochar application rates on soil aggregate stability. (**A**) R > 0.25 (proportion of aggregates > 0.25 mm), (**B**) Mean Weight Diameter (MWD), (**C**) Geometric Mean Diameter (GMD), and (**D**) Fractal Dimension (*D*).

Compared to those of the control (CK), the values of R_0.25_, MWD, and GMD within the same soil layer increased with increasing biochar amendment levels (Figure 2A–C), whereas *D* decreased (Figure 2D). Within the same treatment, R_0.25_, MWD, and GMD decreased with increasing soil depth, whereas the *D* value showed the opposite trend (Figure 2). Specifically, in the 0–10 cm layer, R_0.25_ ranged from 56.86% to 75.40%, MWD from 1.33 to 2.37 mm, and GMD from 0.78 to 1.36 mm; in the 10–20 cm layer, R_0.25_ ranged from 48.04% to 70.69%, MWD from 1.06 to 2.19 mm, and GMD from 0.61 to 1.23 mm; and in the 20–30 cm layer, R_0.25_ ranged from 39.26% to 63.40%, MWD from 0.66 to 1.58 mm, and GMD from 0.39 to 0.93 mm. The D values ranged from 1.53 to 2.27 in the 0–10 cm layer, 1.61 to 2.55 in the 10–20 cm layer, and 1.89 to 2.76 in the 20–30 cm layer. Biochar application significantly increased R_0.25_, MWD, and GMD, while it decreased *D*, indicating that biochar application enhanced macroaggregate proportion and aggregate stability, effectively improving soil structure.

### 3.2. Effects of Biochar Amendment on Fungal and Bacterial Abundance, Gram-Positive/Gram-Negative Ratio, and Fungal/Bacterial Ratio in Soil Aggregates

As presented in Table 1, compared with the control, the fungal PLFA content (fungal abundance) within all aggregate size classes in the same soil layer exhibited a significantly increasing trend with increasing biochar amendment levels, ranked as CK < T1 < T2 < T3 < T5 < T4. Specifically, in the 0–10 cm layer, fungal PLFA content ranged from 2.28 to 12.74 nmol/g; in the 10–20 cm layer, from 2.03 to 11.16 nmol/g; and in the 20–30 cm layer, from 1.19 to 9.87 nmol/g. Across all treatments and soil layers, fungal PLFA content was generally higher in macroaggregates (≥0.25 mm). Within the same treatment, fungal PLFA content in all aggregate size classes decreased significantly with increasing soil depth. The bacterial PLFA content (bacterial abundance) within all aggregate size classes in the same soil layer increased with increasing biochar amendment levels (Table 1). Within the same treatment, the bacterial PLFA content in all aggregate size classes exhibited a significant decreasing trend as soil depth increased. Except for the CK (0–10 cm), the bacterial PLFA content across all treatments and soil layers generally showed an increasing trend with increasing aggregate particle size. Specifically, in the 0–10 cm layer, bacterial PLFA content ranged from 10.28 to 29.12 nmol/g; in the 10–20 cm layer, from 5.78 to 22.64 nmol/g; and in the 20–30 cm layer, from 4.13 to 17.41 nmol/g. Biochar application significantly increased both fungal and bacterial abundances in all aggregate size classes, showing an increasing trend with application rate and a decreasing trend with soil depth.

As shown in Table 2, compared with the control (CK), the PLFA content of Gram-positive bacteria within all aggregate size classes in the same soil layer increased with increasing biochar application rates, reaching a maximum at the T5 treatment. Within the same treatment, the PLFA content of Gram-positive bacteria across all aggregate size classes exhibited a decreasing trend with increasing soil depth. Specifically, in the 0–10 cm layer, the PLFA content of Gram-positive bacteria ranged from 4.75 to 15.01 nmol/g; in the 10–20 cm layer, from 2.70 to 12.25 nmol/g; and in the 20–30 cm layer, from 1.35 to 11.02 nmol/g. The PLFA content of Gram-negative bacteria within all aggregate size classes in the same soil layer exhibited a significant increasing trend with increasing biochar application rates, followed by a decrease at higher application rates, with the order of CK < T1 < T2 < T3 < T5 < T4, peaking at T4. Within the same treatment, the PLFA content of Gram-negative bacteria across all aggregate size classes decreased with increasing soil depth. Specifically, in the 0–10 cm layer, the PLFA content of Gram-negative bacteria ranged from 4.12 to 13.02 nmol/g; in the 10–20 cm layer, from 2.12 to 10.42 nmol/g; and in the 20–30 cm layer, from 1.06 to 8.27 nmol/g.

Across all aggregate size classes, the PLFA content of bacteria was consistently higher than that of fungi, and the PLFA content of Gram-positive bacteria was consistently higher than that of Gram-negative bacteria. Furthermore, most biochar-amended treatments exhibited relatively higher fungal/bacterial ratios (F/B) and Gram-positive/Gram-negative bacterial ratios (G^+^/G^−^) compared with the CK (Table 3). Specifically, in the 0–10 cm layer, the F/B ratio ranged from 0.22 to 0.52, and the G^+^/G^−^ ratio ranged from 1.02 to 1.40; in the 10–20 cm layer, the F/B ratio ranged from 0.26 to 0.56, and the G^+^/G^−^ ratio ranged from 1.05 to 1.69; and in the 20–30 cm layer, the F/B ratio ranged from 0.29 to 0.65, and the G^+^/G^−^ ratio ranged from 1.02 to 2.14.

### 3.3. Contents of Carbon Fractions in Water-Stable Soil Aggregates and Their Contributions to Total Soil Organic Carbon Under Different Biochar Application Rates

Compared to those of the CK, the contents of OC, ROC, EOC, POC, MBC, DOC and BC within the aggregates of different size classes in the same soil layer exhibited an upward trend with increasing biochar amendment levels, and MBC and DOC contents peaked slightly in the T4 treatment (Table 4, Table 5, Table 6, Table 7, Table 8, Table 9 and Table 10). Within the same treatment, the OC, ROC, EOC, POC, MBC, DOC, and BC contents in all aggregate size classes exhibited a significant decreasing trend with increasing soil depth (*p* < 0.05). Specifically, in the 0–10 cm layer, the contents of OC, ROC, EOC, POC, MBC, DOC, and BC ranged from 6.27 to 23.52 g/kg, 1.54 to 8.51 g/kg, 1.48 to 7.52 g/kg, 1.23 to 8.19 g/kg, 98.34 to 323.52 mg/kg, 15.33 to 38.75 mg/kg, and 1.90 to 11.15 g/kg, respectively. In the 10–20 cm layer, they ranged from 4.46 to 17.79 g/kg, 0.64 to 6.21 g/kg, 1.50 to 6.45 g/kg, 0.95 to 4.85 g/kg, 86.08 to 230.81 mg/kg, 9.33 to 23.69 mg/kg, and 1.31 to 8.18 g/kg, respectively. In the 20–30 cm layer, they ranged from 2.32 to 12.56 g/kg, 0.34 to 1.95 g/kg, 0.70 to 4.70 g/kg, 0.51 to 3.03 g/kg, 37.82 to 170.07 mg/kg, 3.08 to 18.21 mg/kg, and 0.82 to 5.85 g/kg, respectively.

Across all treatments and soil layers, the content of these carbon fractions was generally increased in large macroaggregates (>2 mm) and small macroaggregates (2–0.25 mm). The trends in the contribution rates differed from those of content. In the same soil layer, the contribution rates of OC, ROC, EOC, POC, MBC, DOC, and BC from macroaggregates (≥0.25 mm) generally increased with increasing biochar application rates, whereas those from microaggregates (<0.25 mm) decreased. Generally, under the same treatment, the contribution rates of most OC, EOC, POC, MBC, DOC, and BC from macroaggregates (≥0.25 mm) slightly tended to decrease with soil depth, while those from microaggregates (<0.25 mm) increased; the contribution rate of ROC across all aggregate size classes tended to decrease with soil depth. Specifically, in the 0–10 cm layer, the contribution rates of OC, ROC, EOC, POC, MBC, DOC, and BC ranged from 8.26% to 51.44%, 2.80% to 18.60%, 2.60% to 16.44%, 2.73% to 17.92%, 0.10% to 0.71%, 0.01% to 0.10%, and 3.50% to 24.39%, respectively. In the 10–20 cm layer, they ranged from 10.82% to 49.33%, 2.41% to 17.21%, 2.58% to 17.89%, 1.89% to 14.14%, 0.14% to 0.67%, 0.01% to 0.07%, and 4.61% to 22.67%, respectively. In the 20–30 cm layer, they ranged from 14.12% to 46.50%, 2.28% to 7.54%, 4.41% to 17.39%, 3.15% to 11.01%, 0.19% to 0.66%, 0.02% to 0.07%, and 4.78% to 21.66%, respectively. Biochar application enhanced the OC, ROC, EOC, POC, MBC, DOC, and BC contents in all aggregate size classes, and their contribution rates to macroaggregates increased with increasing amendment levels.

Across the 0–30 cm soil profile, the average contribution rates of OC, ROC, EOC, POC, MBC, DOC, and BC from macroaggregates were significantly higher than those from microaggregates. Additionally, in the 0–10 cm soil layer, ROC and BC contents and their contribution rates to total SOC were consistently higher than those of the labile carbon fractions within all aggregate size classes. This indicates that biochar may promote the accumulation of organic carbon to a certain extent through the formation of macroaggregates and an increase in inert carbon.

### 3.4. Relationships Among Aggregate Size Classes, Aggregate Stability, Aggregate-Associated Fungi and Bacteria, and Organic Carbon Fractions Under Biochar Application

**Figure 3:**
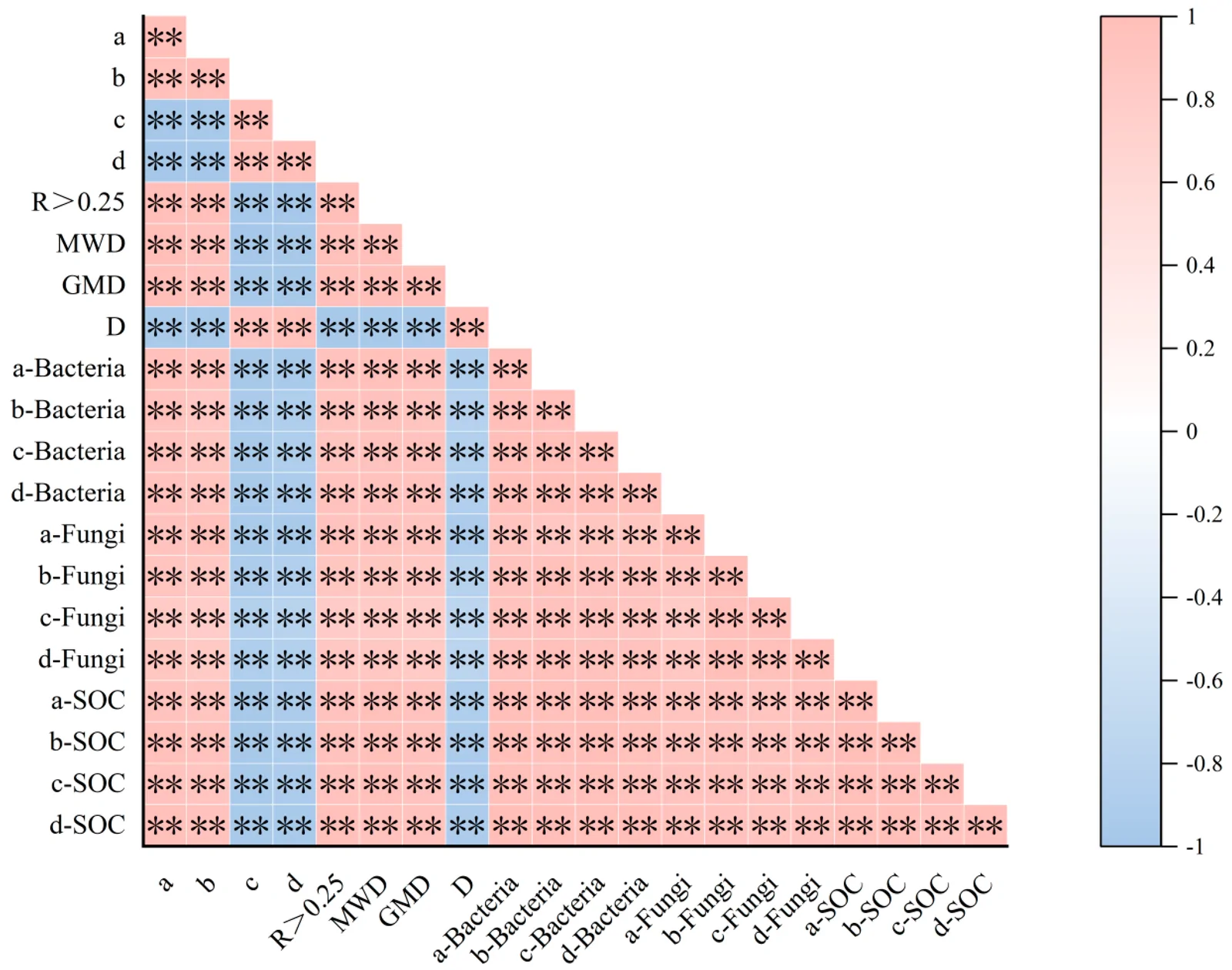
Pearson correlation heatmap among aggregate size classes, aggregate stability, aggregate-associated fungi and bacteria, and aggregate organic carbon. The code ‘a’ denotes > 2 mm aggregates, ‘b’ denotes 2–0.25 mm aggregates, ‘c’ denotes 0.25–0.053 mm aggregates, and ‘d’ denotes < 0.053 mm aggregates. Prefixes (a, b, c, d) attached to other variables indicate microorganisms or organic carbon associated with the corresponding aggregate size class. Red denotes a positive correlation between the two variables, while blue indicates a negative correlation; the darker the color, the stronger the correlation. ** indicates *p* < 0.01. The same as below.

**Figure 4:**
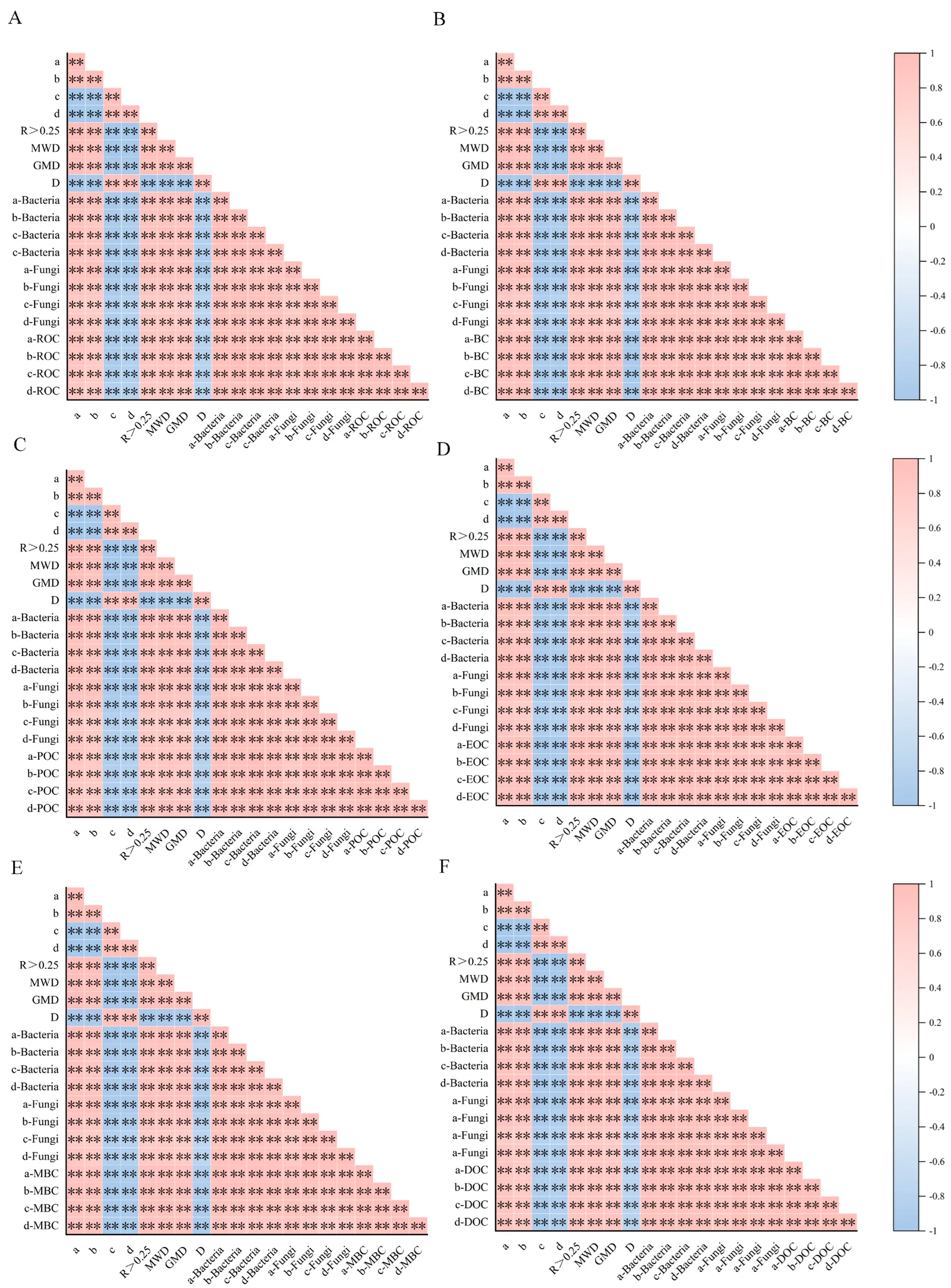
Pearson correlation heatmaps among aggregate size fractions, aggregate stability, aggregate-associated fungi and bacteria, and carbon fractions. Panels (**A**–**F**) correspond to the carbon fractions: ROC, BC, POC, EOC, MBC, and DOC, respectively.

Correlation analysis results (Figure 3 and Figure 4) indicated significant positive correlations (*p* < 0.01) among macroaggregates (a + b), aggregate stability (R_0.25_, MWD, and GMD), and aggregate-associated fungal and bacterial abundance. These variables also exhibited significant positive correlations (*p* < 0.01) with organic carbon and its fractions within all aggregate size classes. However, all of them demonstrate significant negative correlations with microaggregates (c + d) and *D* (*p* < 0.01). Macroaggregates showed positive correlations with the corresponding microbial abundances and carbon fractions, whereas microaggregates showed negative correlations.

**Figure 5:**
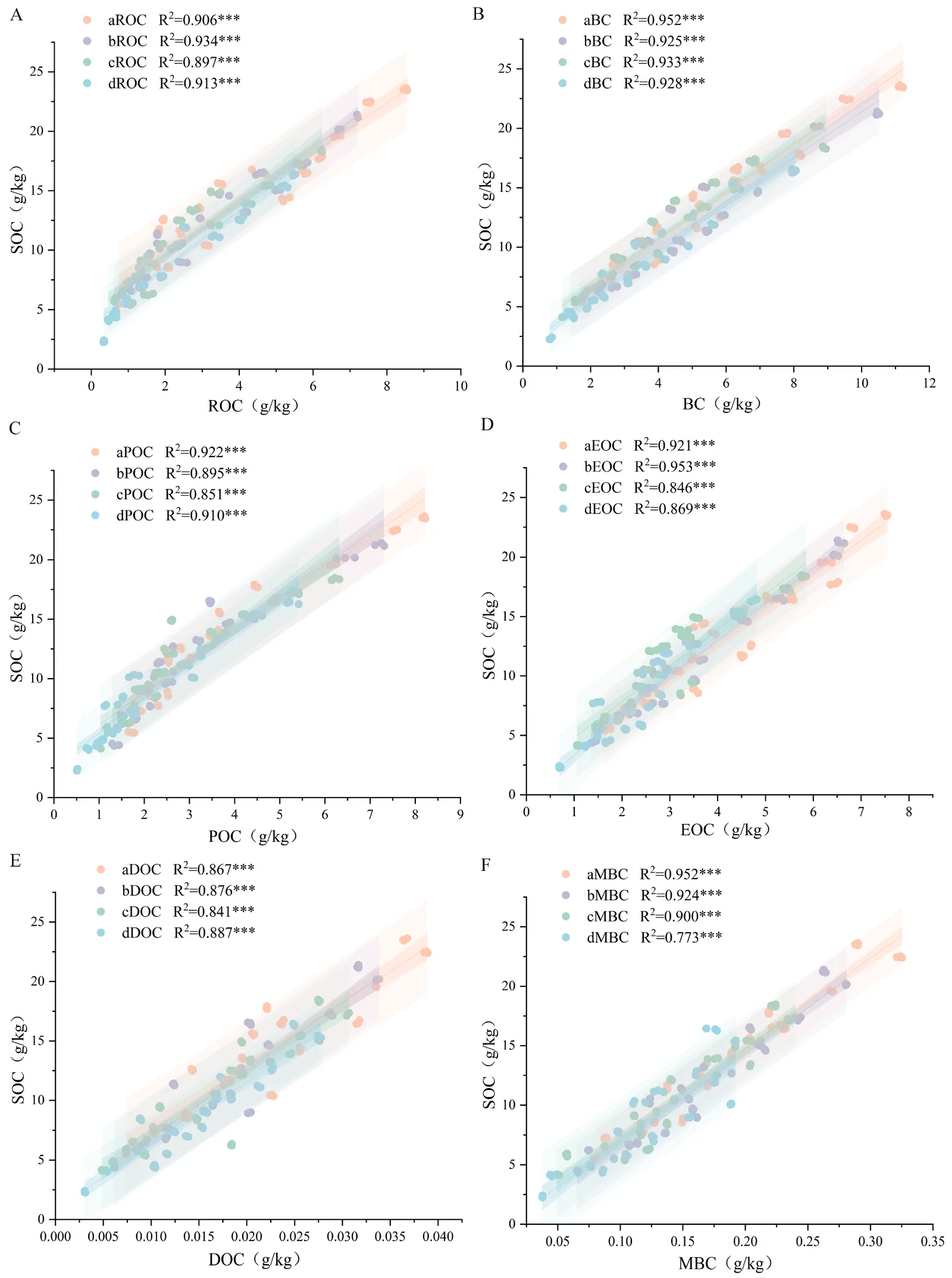
Regression analysis between organic carbon and carbon fractions across aggregate size classes. Panels (**A**,**B**) represent recalcitrant carbon fractions (ROC and BC), while (**C**–**F**) represent labile carbon fractions (POC, EOC, DOC, and MBC). *** denote statistical significance at *p* < 0.001.

Linear regression showed a close coupling relationship between aggregated organic carbon and the corresponding recalcitrant carbon (ROC and BC) and labile carbon (POC, EOC, DOC, and MBC) components in all aggregate particle sizes (Figure 5). The main carbon components of aggregates with a coefficient of determination >0.90 were BC, MBC, ROC, and EOC. This suggests that aggregate particle size influences the coupling relationship between carbon fractions and organic carbon, indicating that biochar application may affect SOC stabilization and turnover by altering carbon fractions within different aggregate size classes.

**Figure 6:**
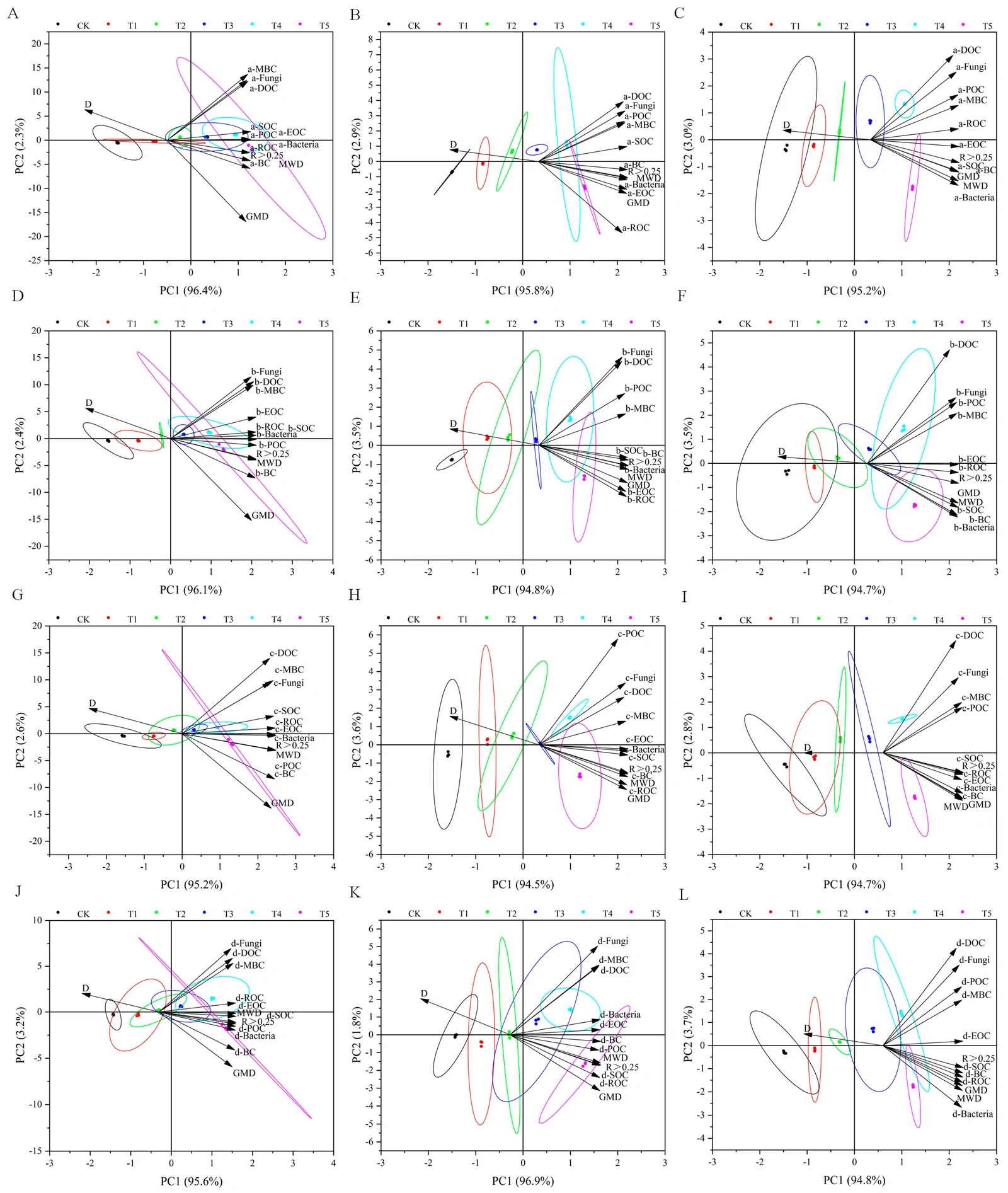
Principal component analysis (PCA) of aggregate stability, aggregate-associated fungi and bacteria, and carbon fractions within aggregates across different soil layers. Panels (**A**–**C**) represent PCA results for >2 mm aggregates in 0–10, 10–20, and 20–30 cm soil layers, respectively; (**D**–**F**) for 2–0.25 mm aggregates; (**G**–**I**) for 0.25–0.053 mm aggregates; and (**J**–**L**) for < 0.053 mm aggregates.

The PCA results (Figure 6) showed that across all aggregate size classes, the first two principal components (PC1 and PC2) cumulatively explained more than 98% of the total variance (PC1: 95.2–96.4%; PC2: 2.3–3.5%), indicating that these two components adequately captured the major sources of variation. PC1 was primarily associated with the overall improvement of soil properties, with high positive loadings (>0.26) for most variables including SOC, POC, bacterial PLFA, R_0.25_, MWD, and BC, while *D* consistently showed negative loadings (approximately −0.28) across all aggregate size classes. These variables jointly drove the separation among treatments. In contrast, PC2 was predominantly driven by GMD, fungal PLFA, MBC, and DOC. GMD consistently exhibited strong negative loadings (−0.56 to −0.57), while fungal PLFA (0.42–0.54), MBC (0.37–0.46), and DOC (0.40–0.54) showed positive loadings on PC2. In the 0–10 cm layer, POC, EOC, and ROC within the aggregates were most closely related to SOC. In the 10–20 cm layer, SOC exhibited the highest correlation with BC in the >2 and 2–0.25 mm aggregates, with EOC in the 0.25–0.053 mm aggregates, and with ROC in the <0.053 mm aggregates. In the 20–30 cm layer, SOC was most strongly correlated with BC in the >2 and 2–0.25 mm aggregates, with ROC and EOC in the 0.25–0.053 mm aggregates, and with BC and ROC in the <0.053 mm aggregates.

Across all soil layers, aggregate-associated fungi showed weak correlations with recalcitrant carbon fractions (BC and ROC). However, they were highly correlated with the labile carbon fractions (MBC and DOC) in the 0–10 cm layer and the labile fractions (MBC, DOC, and POC) in the 10–30 cm layers. In contrast, bacteria within aggregates across all layers were closely associated with the corresponding aggregate stability indices (R_0.25_, MWD, and GMD) and other carbon fractions of the aggregates. Substantial positive correlations were observed among stability indices (MWD, GMD, and R_0.25_), aggregate organic carbon, carbon fractions, and fungal and bacterial abundances, whereas strong negative correlations were observed with the *D*, consistent with the correlation analysis results.

**Figure 7:**
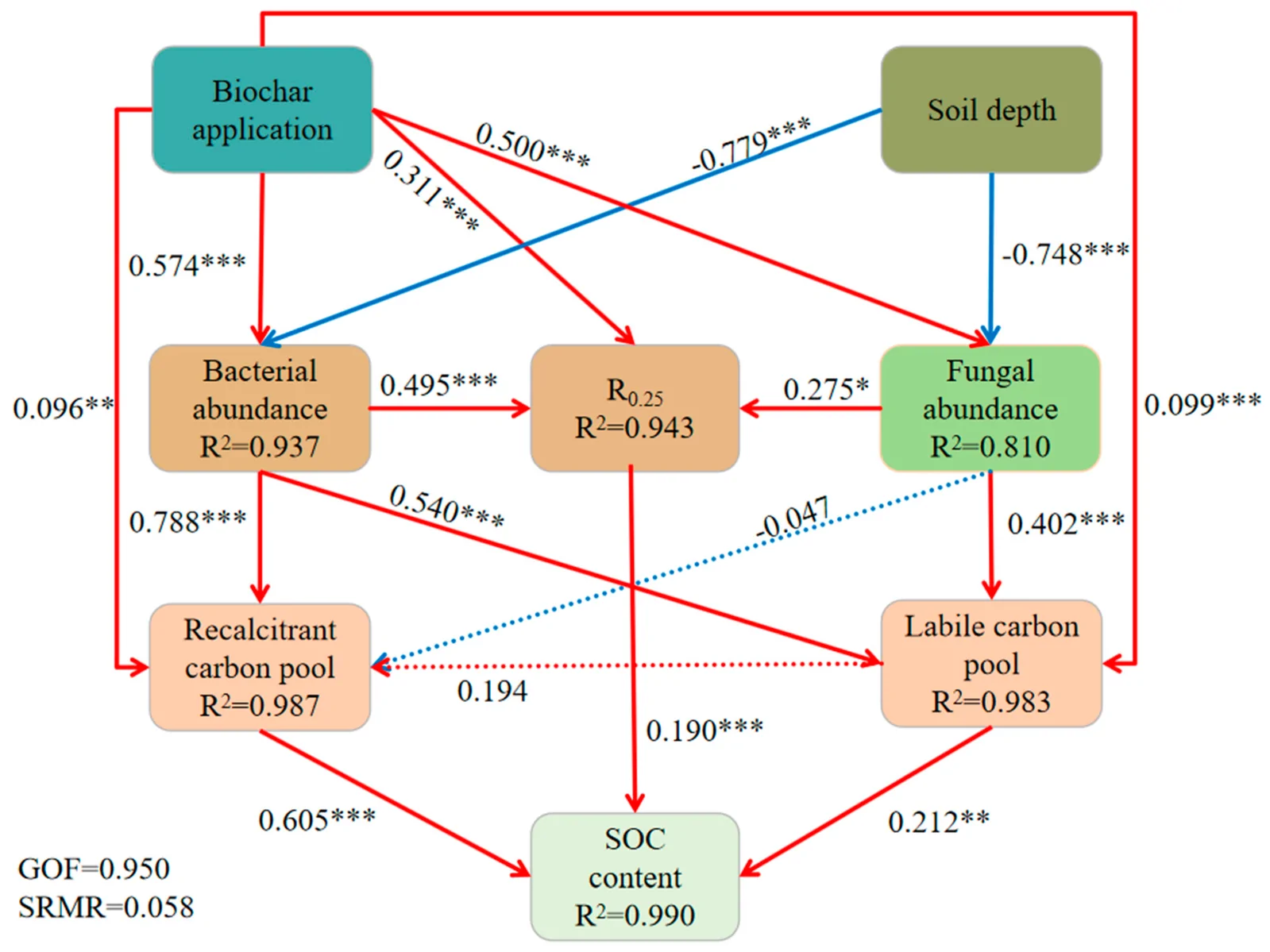
Structural equation model (SEM) illustrating the hypothesized pathways among aggregate stability, aggregate-associated fungal and bacterial abundances, and aggregate organic carbon under biochar application. Red and blue arrows indicate positive and negative effects, respectively. Solid and dashed lines represent significant and non-significant effects, respectively. Numbers adjacent to arrows are standardized path coefficients. R^2^ indicates the proportion of variance explained. GOF represents the Goodness of Fit, and SRMR represents the standardized root mean square residual. Significance levels are indicated as follows: * *p* < 0.05, ** *p* < 0.01, and *** *p* < 0.001.

Figure 6 shows that, with increasing biochar application rates (from CK to T5), the treatment groups shifted from negative to positive scores along the PC1 axis, gradually separating them from CK. T5 showed the highest correlation with the PC1 axis, further indicating that increasing biochar application positively affected aggregate stability, carbon fractions, and microbial communities, and the ameliorative effect gradually intensified. The structural equation modeling (SEM) results (Figure 7) indicated that biochar application and soil depth directly affected aggregate SOC content by mediating fungal and bacterial abundance, which subsequently influenced aggregate stability. Biochar also directly affected aggregate stability, recalcitrant carbon pool, and labile carbon pool. Additionally, fungal and bacterial abundances promoted SOC stabilization by influencing the recalcitrant and labile carbon pools, and fungi did not have a significant effect on the recalcitrant carbon pool.

## 4. Discussion

### 4.1. Response of Soil Aggregates Distribution Patterns and Stability to Biochar Amendment and Application Rate

The incorporation of biochar into soil can improve the soil structure and promote the formation of soil aggregates [1,16,27,63]. Following a seven-year period of biochar amendment, our results indicated that biochar incorporation significantly increased soil macroaggregate proportion (≥0.25 mm), a trend positively correlated with escalating biochar application rates, whereas microaggregate proportion (<0.25 mm) exhibited a reduction (Figure 1). The vertical gradient in aggregate distribution may be partly related to the higher root density and greater litter input in the surface soil layer of *Eucalyptus* plantations, which promote macroaggregate formation through root entanglement and organic matter binding. This may be because the biochar acts as a cementing agent and interacts with clay minerals and organic matter in the soil to promote aggregate formation and stability [64]. Biochar provides microhabitats and carbon sources that enhance soil microbial activity, leading to the production of binding substances, such as exudates and fungal hyphae. These substances reinforce the cementing effects, thereby facilitating the transformation of microaggregates into macroaggregates [23]. Additionally, biochar application significantly increased the MWD, GMD, and R_0.25_ of the soil, while effectively reducing *D* (Figure 2). This indicates a considerable enhancement in the soil erosion resistance and aggregate stability, findings that align with prior investigations [36,65].

Several short-term experiments have not observed the impacts of biochar on aggregate stability [66,67]. Notably, in understory environments lacking tillage and root exudates, biochar amendment reduces macroaggregate proportion and aggregate stability [35]. This indicates that macroaggregate formation and stability do not rely solely on biochar but may be synergistically influenced by root exudates, hyphae, and fungi. This aligns with the hierarchical development model of aggregates [8], which posits that the formation of macroaggregates relies on plant roots and fungal hyphae to bind and entangle < 0.053 mm soil aggregates. Although we did not measure the biomass of *Eucalyptus* roots, we observed that the application of biochar after 7 years increased the fungal PLFA content in soil aggregates.

Improvement in soil aggregate stability is frequently accompanied by an increase in SOC. As a vital cementing agent, organic carbon promotes the cohesion of minerals and clay particles to generate macroaggregates. Stable aggregates inhibit biotic and abiotic decomposition processes, thereby providing physical protection for SOC, which enhances soil carbon accumulation and the stability of soil structure [9,55]. Additionally, we observed that macroaggregate content, R_0.25_, MWD, and GMD exhibited a marked decline with increasing soil depth, whereas microaggregate content and *D* values demonstrated a significant upward trend (Figure 2). This trend may be attributed to restricted oxygen supply, reduced microbial activity, and lower SOC content at greater depths. Additionally, deep soil exhibits low root density and minimal litter input. Consequently, the cohesive forces among soil particles are attenuated, and the lack of hyphal entanglement makes the formation or maintenance of macroaggregates challenging, resulting in a structure dominated by fine microaggregates [63,68,69]. This indicates that the surface soil layer (0–10 cm) exhibits increased sensitivity to biochar input, which facilitates the formation of macroaggregates and improves aggregate stability.

### 4.2. Responses of Microbial Communities in Soil Aggregates to Biochar Application

Soil aggregates are the primary sites for microbial activity and nutrient cycling [70]. Spatial heterogeneity among different aggregate size classes creates distinct ecological niches for microbial communities [71,72,73]. Owing to their porous structure and labile components, biochar inputs provide habitats and nutrients for microbial growth, thereby influencing the distribution of microbial communities across distinct aggregate size fractions [24,74]. Our results indicated that, compared to those of CK, biochar application significantly increased fungal and bacterial abundances within all aggregate size classes, which typically increased with application rates. This suggests that biochar addition stimulates microbial growth and reproduction; however, excessive biochar may exert an inhibitory effect on fungal abundance. This phenomenon may be related to the propensity of fungi for acidic conditions [75], as biochar application is known to influence soil pH. The observed increase in fungal abundance at moderate biochar application rates and the subsequent decline at the highest rate (T5) could reflect pH-related effects or shifts in microbial competitive interactions; however, as soil pH was not directly measured in the present study, this interpretation remains speculative and requires further investigation.

Soil bacterial and fungal abundances exhibited an increasing trend with increasing aggregate particle size, identifying macroaggregates as “functional hotspots” in the soil. Macroaggregates (>0.25 mm) have a relatively open structure and are rich in fresh plant residues and labile organic carbon, thereby providing sufficient substrate and oxygen for microbial metabolism [39,76,77]. By contrast, microaggregates (<0.25 mm) are characterized by restricted pore spaces and physical barriers that limit the rapid exchange of oxygen and water, hindering microbial colonization and resulting in reduced abundance [39]. This further underscores the importance of macroaggregates in providing a suitable survival environment that facilitates microbial growth and reproduction [5]. Additionally, we observed that the PLFA abundance of bacteria consistently exceeded that of fungi across all treatments, soil layers, and aggregate size classes. This indicates that bacteria are the dominant components of the soil microbial community, exhibiting a broader adaptation range to environmental changes and an increased efficiency in utilizing the microhabitats and nutrient pools provided by biochar for turnover [78]. Beyond simple shifts in abundance, our results suggest that biochar addition may fundamentally reshape microbial survival strategies. The observed increase in the abundance of major microbial groups (as indicated by PLFAs) likely reflects a shift favoring copiotrophic taxa over oligotrophic taxa due to increased nutrient availability [30,78]. Recent studies highlight that such a community reconfiguration—specifically the enrichment of resource-acquisitive, broad-niche microbial groups—serves as a leading indicator for the potential accumulation of microbial-derived carbon and enhanced carbon sequestration efficiency [21,30]. Studies have shown a positive correlation between the PLFA content and microbial necromass in biochar-amended soils [79], suggesting that the observed increase in microbial biomass may contribute to the accumulation of stable carbon pools. In the present study, biochar application significantly increased the PLFA contents of both Gram-positive and Gram-negative bacteria, which is consistent with the findings of Ameloot et al. [80] in a sandy loam soil amended with willow-derived biochar. This may be attributed to the greater environmental adaptability of Gram-positive bacteria compared to Gram-negative bacteria, enabling them to utilize complex carbon sources more efficiently in soil [81].

The fungal/bacterial (F/B) ratio is a critical indicator for measuring the relative changes in quantity and abundance, indicating the characteristics of the soil microbial community structure [82]. Owing to the stronger ability of fungi to degrade complex C polymers, an increase in soil (F/B) facilitates the conversion of SOC to refractory components, thereby altering the SOC fraction [83]. Fungal-dominated communities promote soil aggregate formation through the formation of hyphal networks [84]. The increased F/B ratio indicates that biochar application improved the soil microbial community structure, creating conditions that were relatively more favorable for fungal growth and colonization. This shift facilitates the more efficient utilization of complex carbon substrates, promotes the formation and stabilization of soil aggregates, and contributes to the stable accumulation of SOC [85,86,87,88,89].

### 4.3. Effects of Biochar Amendment on Aggregate-Associated OC and Its Carbon Fractions

Compared with the CK, biochar application significantly enhanced the OC content in the aggregates across all particle size fractions, and showed a positive correlation with the biochar amendment levels (Table 4). These results are consistent with prior research [34,90] and may be attributed to the C-rich nature of biochar itself, which directly increases the soil OC content following application [91]. Organic carbon is a crucial cementing agent for soil aggregate formation, which enhances soil particle cohesion and facilitates aggregate formation and stability. Conversely, aggregates provide optimal storage sites for OC, reducing its decomposition and loss while promoting internal accumulation, indicating a close relationship with each other [7,92,93].

Although previous studies demonstrated a significant positive relationship between aggregates and OC content within plantation soils [94], this was not entirely consistent with the results of our correlation analysis (Figure 3). In this study, a positive correlation was observed between the macroaggregate proportion and macroaggregate OC content, whereas the microaggregates proportion was negatively correlated with microaggregate OC content. This phenomenon is likely due to the impact of biochar amendment on aggregate formation and turnover processes. Biochar facilitates the conversion of microaggregates into macroaggregates, thereby reducing microaggregate proportion and enhancing the enrichment and physical protection of OC within macroaggregates [52,76].

Labile SOC fractions (EOC, POC, MBC, and DOC) regulate soil nutrient availability, and are directly involved in the soil carbon cycle [95], acting as sensitive indicators of dynamic changes in the soil carbon pool [96]. Conversely, the recalcitrant SOC fractions (ROC and BC) are critical for augmenting soil carbon pool and stability [97]. Our results demonstrate that biochar amendment significantly elevated the contents of ROC, EOC, POC, MBC, DOC, and BC within all aggregate size classes. While these fraction contents increased with the biochar application rate, MBC and DOC levels reached their maximum values at T4 (Table 5, Table 6, Table 7, Table 8, Table 9 and Table 10). The increases in ROC and BC contents may be attributed to the conversion of applied biochar into soil black carbon [43] or to microbial processes [98]. The highly aromatic molecular structure of biochar directly contributed to the increased stable carbon content in the soil. Biochar can decompose into active fine particulate solids owing to its loose and porous structure, thereby promoting the accumulation of labile OC fractions, which increase with increasing application rates [99,100]. Additionally, biochar enhances soil microbial activity, accelerating the microbial conversion of plant residues into labile carbon fractions, thereby increasing labile carbon content in the soil [12].

DOC is highly bioavailable and serves as a vital substrate for microbial proliferation, impacting the availability and transport of nutrients within ecosystems [101,102]. The observed increases in DOC and MBC may be attributed to the biochar providing carbon sources, energy, and sites for microbial growth, thereby boosting microbial activity and facilitating the decomposition of insoluble substances [12,103]. In this study, biochar increased the pH, and fungi were closely correlated with DOC and MBC across all aggregate size fractions and soil layers (Figure 6). An increased pH may inhibit fungal growth and reproduction [75], leading to a decline in DOC and MBC. This suggests that in biochar-amended soils, fungi may influence SOC stabilization and turnover, primarily by regulating the labile carbon fractions (Figure 7). In the 0–10 cm soil layer, the recalcitrant carbon fraction contents (ROC and BC) within all aggregate size classes were higher than those of other fractions and showed a positive growth trend with the increase in biochar amendment (Table 5, Table 6, Table 7, Table 8, Table 9 and Table 10). This indicates that biochar amendment enhances the stabilization of OC in surface soil. As a fast-growing tree species with high litter production and extensive root systems, *Eucalyptus* may directly contribute to the observed carbon accumulation through continuous litterfall and root-derived carbon inputs, particularly in the surface soil layer where root density is highest.

The contribution rates of various carbon fractions within soil aggregates are jointly determined by the structural characteristics of the aggregates and the distribution and content of carbon fractions across different particle sizes [104]. In the present study, biochar application notably augmented the relative contributions of SOC, EOC, POC, MBC, DOC, ROC, and BC derived from macroaggregates (>0.25 mm) (Table 4, Table 5, Table 6, Table 7, Table 8, Table 9 and Table 10). Within macroaggregates, the contribution of organic carbon accounted for the largest proportion, which was primarily attributed to biochar addition providing sufficient carbon sources for microbial activity; the unique pore structure of macroaggregates provides a more suitable habitat for microorganisms, facilitating the substantial fixation and storage of exogenous carbon within these structures [8]. The OC content in soil aggregates escalates with the augmentation of particle size, and macroaggregates contain increased amounts of carbon [105]. Owing to the increased content and larger mass proportion of macroaggregates, their contribution rate to organic carbon was consequently increased, which is consistent with previous studies [106].

The relative contribution rates of the carbon fractions from macroaggregates were significantly higher than those from the other aggregate size classes. These results indicated that the carbon fractions were mainly enriched in macroaggregates [76]. The relatively open structure of macroaggregates provides a favorable habitat for microorganisms, promoting the decomposition and transformation of carbon fractions and increasing their contents within macroaggregates. Recalcitrant carbon fractions are crucial components for aggregate stability, and their high contribution rates promoted the formation of more stable and durable aggregate structures [55], thereby optimizing soil aggregate structure and enhancing the stability of the soil carbon pool. Thus, we hypothesized that biochar increases microbial biomass, which subsequently results in the accumulation of microbial residues and, consequently, elevates the recalcitrant carbon content.

### 4.4. Limitations and Future Prospects

While this study provides valuable insights, several limitations remain. First, the effects of biochar application at different rates on the storage, composition, and underlying mechanisms of native soil organic carbon (n-SOC) dynamics in plantation soil ecosystems remain unclear. Future research can use the “water flotation method”, improved “combustion loss method”, and isotopic signatures (δ^13^C) to compare and quantify residual biochar in soil. While the biochar demonstrates beneficial effects, it may also possess potential limitations, including possible alkalization, nutrient fixation, and environmental risks associated with high application rates. We will continue to conduct further detailed observations in subsequent experiments. Second, soil aggregates play a critical regulatory role in improving microbial community structure, but further in-depth analysis is needed on the mechanisms of their effects on different microorganisms. We acknowledge that PLFA is a viable method, but it also has limitations: while PLFA can provide information on microbial biomass, it cannot reveal taxonomic composition or functional diversity and requires further in-depth investigation using high-throughput sequencing. Additionally, for different soil ecosystem management regimes, the carbon sequestration potential of diverse ecosystems can be assessed following long-term biochar application, and the underlying mechanisms driving variations in total soil organic carbon (t-SOC) and n-SOC can be explored through the examination of OC fractions, soil aggregates, and microorganisms. Therefore, more detailed research is needed in the practical application of agriculture and forestry.

## 5. Conclusions

The 7-year trials demonstrated that biochar amendment effectively increased SOC and its components in *Eucalyptus* plantation soils, with recalcitrant carbon as the primary contributor. Biochar application significantly facilitated the conversion of microaggregates into macroaggregates, enhanced the physical stability of the soil structure, and increased bacterial and fungal abundances across all aggregate size classes. Biochar application was associated with elevated contents of both recalcitrant (ROC and BC) and labile (EOC, POC, MBC, and DOC) carbon fractions, with relatively inert carbon components dominating and the most pronounced increments observed in macroaggregates. Based on the structural equation, it can be inferred that biochar application might enhance the long-term carbon sequestration potential of Eucalyptus plantation soil through a synergistic mechanism involving “aggregate structure optimization—microbial community regulation—carbon fraction enrichment.” This mechanism might provide physical protection for OC and facilitated chemical sequestration through the action of the microbial carbon pump, to a certain degree. The application rates of 4% and 6% produced the largest responses for most measured indicators. Our findings provide a theoretical and technical basis for synergistically enhancing the carbon stabilization and fertility of plantation soils.

## Figures and Tables

**Table 1:** Effects of different biochar application rates on fungal and bacterial PLFA contents in soil aggregates.

Soil Depth (cm)	Treatment	Fungal PLFA (nmol/g)				Bacterial PLFA (nmol/g)			
		>2 mm	2–0.25 mm	0.25–0.053 mm	<0.053 mm	>2 mm	2–0.25 mm	0.25–0.053 mm	<0.053 mm
0–10	CK	6.73 fA	5.55 fA	2.28 fA	4.27 fA	15.99 fA	15.05 fA	10.28 fA	11.42 fA
	T1	9.70 eA	8.94 eA	6.77 eA	6.05 eA	19.60 eA	17.82 eA	13.54 eA	12.47 eA
	T2	10.60 dA	9.77 dA	7.70 dA	6.66 dA	22.65 dA	21.19 dA	16.54 dA	14.35 dA
	T3	11.65 cA	11.28 cA	9.79 cA	8.17 cA	24.91 cA	22.89 cA	18.86 cA	16.24 cA
	T4	12.74 aA	12.14 aA	10.43 aA	9.85 aA	27.18 bA	25.22 bA	22.65 bA	19.20 bA
	T5	12.25 bA	11.47 bA	10.11 bA	8.43 bA	29.12 aA	27.05 aA	24.35 aA	21.29 aA
10–20	CK	4.31 fB	3.40 fB	2.12 fB	2.03 fB	11.94 fB	10.16 fB	8.01 fB	5.78 fB
	T1	6.24 eB	5.12 eB	3.13 eB	2.77 eB	13.65 eB	12.15 eB	10.15 eB	7.11 eB
	T2	8.21 dB	5.69 dB	3.86 dB	3.42 dB	16.69 dB	13.64 dB	12.18 dB	10.20 dB
	T3	10.06 cB	6.86 cB	4.41 cB	4.42 cB	18.08 cB	15.80 cB	13.65 cB	12.23 cB
	T4	11.16 aB	8.86 aB	5.41 aB	5.55 aB	20.04 bB	16.86 bB	15.16 bB	13.64 bB
	T5	10.65 bB	7.20 bB	4.69 bB	4.84 bB	22.64 aB	18.72 aB	16.11 aB	14.65 aB
20–30	CK	2.67 eC	2.42 fC	1.51 fC	1.19 eC	8.50 fC	7.20 fC	5.27 fC	4.13 fC
	T1	4.36 dC	3.37 eC	2.16 eC	1.86 dC	10.17 eC	8.04 eC	6.28 eC	5.39 eC
	T2	6.25 cC	4.08 dC	2.93 dC	2.45 cC	11.78 dC	9.10 dC	7.23 dC	6.28 dC
	T3	8.20 bC	4.83 cC	3.11 cC	3.13 bC	13.24 cC	10.41 cC	8.06 cC	7.28 cC
	T4	9.87 aC	5.59 aC	3.39 aC	3.54 aC	15.13 bC	12.01 bC	9.65 bC	8.23 bC
	T5	8.34 bC	5.13 bC	3.25 bC	3.02 bC	17.41 aC	13.87 aC	10.85 aC	10.18 aC

Significant differences across treatments within the same soil layer are denoted by different lowercase letters (*p* < 0.05), and significant differences across soil layers under the same treatment are denoted by distinct uppercase letters (*p* < 0.05). The same applies hereinafter.

**Table 2:** Effects of different biochar application rates on PLFA contents of Gram-positive and Gram-negative bacteria in soil aggregates.

Soil Depth (cm)	Treatment	Gram-Positive Bacteria (nmol/g)				Gram-Negative Bacteria (nmol/g)			
		>2 mm	2–0.25 mm	0.25–0.053 mm	<0.053 mm	>2 mm	2–0.25 mm	0.25–0.053 mm	<0.053 mm
0–10	CK	8.36 fA	7.33 fA	4.75 fA	5.65 fA	7.95 eA	6.61 fA	4.12 fA	5.15 fA
	T1	10.31 eA	10.00 eA	7.35 eA	7.13 eA	10.08 dA	9.17 eA	6.16 eA	6.02 eA
	T2	12.28 dA	11.24 dA	8.43 dA	8.10 dA	11.90 cA	10.76 cA	7.64 dA	7.16 dA
	T3	13.20 cA	11.42 cA	10.14 cA	9.23 cA	12.13 bA	10.92 bA	9.14 bA	8.67 bA
	T4	14.12 bA	12.94 bA	11.77 bA	10.57 bA	13.02 aA	11.96 aA	10.64 aA	9.76 aA
	T5	15.01 aA	13.59 aA	12.43 aA	11.31 aA	12.01 bcA	10.44 dA	8.88 cB	8.29 cA
10–20	CK	5.39 fB	4.77 fB	3.42 fB	2.70 fB	5.10 fB	4.06 eB	3.42 eB	2.12 fB
	T1	7.24 eB	6.37 eB	4.39 eB	4.13 eB	6.30 eB	5.64 dB	4.05 dB	3.56 eB
	T2	8.65 dB	7.28 dB	6.94 dB	6.10 dB	7.68 dB	6.95 cB	5.03 bB	4.59 cB
	T3	9.79 cB	8.56 cB	7.07 cB	6.43 cB	8.96 bB	8.10 bB	5.13 bB	5.01 bB
	T4	11.70 bB	9.6 bB	7.66 bB	6.94 bB	10.42 aB	8.76 aB	5.75 aB	5.27 aB
	T5	12.25 aB	10.28 aB	8.16 aB	7.36 aB	8.42 cB	7.99 bcB	4.90 cA	4.36 dB
20–30	CK	3.71 fC	2.39 fC	1.76 eC	1.35 fC	3.28 fC	2.00 fC	1.31 fC	1.06 eC
	T1	5.13 eC	4.16 eC	2.18 dC	2.07 eC	4.46 eC	4.06 eC	2.03 eC	1.83 dC
	T2	6.34 dC	4.60 dC	4.36 cC	3.84 dC	5.58 dC	4.35 dC	2.41 dC	2.22 cC
	T3	8.15 cC	5.34 cC	4.48 cC	4.01 cC	7.16 bC	5.10 bC	2.91 cC	2.34 bC
	T4	10.06 bC	5.96 bC	4.86 bC	4.11 bC	8.27 aC	5.40 aC	3.40 aC	3.13 aC
	T5	11.02 aC	6.71 aC	5.38 aC	4.59 aC	6.89 cC	4.87 cC	3.13 bC	2.14 cdC

**Table 3:** Effects of biochar amendment on the ratios of Gram-positive to Gram-negative bacteria and fungi to bacteria in soil aggregates.

Soil Depth (cm)	Treatment	Fungal/Bacterial Ratio (F/B)				Gram-Positive/Gram-Negative Bacteria Ratio (G^+^/G^−^)			
		>2 mm	2–0.25 mm	0.25–0.053 mm	<0.053 mm	>2 mm	2–0.25 mm	0.25–0.053 mm	<0.053 mm
0–10	CK	0.42 cA	0.37 eA	0.22 eC	0.37 eA	1.05 cA	1.11 bB	1.15 bcB	1.10 cB
	T1	0.50 aA	0.50 aA	0.50 bA	0.49 bA	1.02 cB	1.09 bA	1.19 bA	1.19 bB
	T2	0.47 bC	0.46 cA	0.47 cA	0.46 cA	1.03 cB	1.04 cA	1.10 cC	1.13 bcC
	T3	0.47 bC	0.49 abA	0.52 aA	0.50 aA	1.09 bA	1.05 cA	1.11 cC	1.06 cC
	T4	0.47 bC	0.48 bB	0.46 cA	0.51 aA	1.08 bcB	1.08 bcA	1.11 cC	1.08 cB
	T5	0.42 cB	0.42 dA	0.42 dA	0.40 dA	1.25 aC	1.30 aB	1.40 aB	1.36 aC
10–20	CK	0.36 dB	0.33 dB	0.26 dB	0.35 cB	1.06 cA	1.18 bA	1.17 cB	1.28 cA
	T1	0.46 cB	0.42 bB	0.31 bC	0.39 bB	1.15 bA	1.13 cA	1.08 dB	1.16 dA
	T2	0.49 bB	0.42 bB	0.32 bC	0.34 dC	1.13 bcA	1.05 eA	1.38 bB	1.33 bB
	T3	0.56 aB	0.43 bC	0.32 bC	0.36 cC	1.09 cA	1.06 eA	1.38 bB	1.28 cB
	T4	0.56 aB	0.53 aA	0.36 aB	0.41 aC	1.12 bcB	1.10 dA	1.33 bB	1.32 bA
	T5	0.47 cA	0.38 cB	0.29 cB	0.33 dB	1.45 aB	1.29 aB	1.67 aA	1.69 aB
20–30	CK	0.31 fC	0.34 eB	0.29 eA	0.29 dC	1.13 cA	1.19 bA	1.34 dA	1.27 dA
	T1	0.43 eC	0.42 cB	0.34 cB	0.35 cC	1.15 cA	1.02 eB	1.08 eB	1.13 eA
	T2	0.53 cA	0.45 bA	0.40 aB	0.39 bB	1.14 cA	1.06 dA	1.81 aA	1.73 bA
	T3	0.62 bA	0.46 aB	0.39 bB	0.43 aB	1.14 cA	1.05 deA	1.54 cA	1.71 bA
	T4	0.65 aA	0.47 aC	0.35 cB	0.43 aB	1.22 bA	1.10 cA	1.43 dA	1.31 cA
	T5	0.48 dA	0.37 dC	0.30 dB	0.30 dC	1.60 aA	1.38 aA	1.72 bA	2.14 aA

**Table 4:** Contents of organic carbon (OC) in soil aggregates of varying size classes and their contribution rates to total organic carbon under different biochar application rates.

Soil Depth (cm)	Treatment	OC Content (g/kg)				OC Contribution Rates (%)			
		>2 mm	2–0.25 mm	0.25–0.053 mm	<0.053 mm	>2 mm	2–0.25 mm	0.25–0.053 mm	<0.053 mm
0–10	CK	10.40 fA	8.98 fA	6.27 fA	7.76 fA	44.19 cA	22.99 dA	12.81 bC	24.22 aA
	T1	14.30 eA	13.19 eA	12.12 eA	10.23 eA	43.02 dA	26.05 cA	14.80 aC	18.97 bC
	T2	16.56 dA	15.06 dA	13.90 dA	11.15 dA	44.37 cA	26.62 cA	12.92 bC	17.23 cB
	T3	19.56 cA	17.25 cA	15.43 cA	12.63 cA	48.69 bA	29.58 bA	10.31 cC	15.59 dC
	T4	22.45 bA	20.14 bA	17.24 bA	15.36 bA	49.56 bA	30.23 bA	8.87 dC	14.69 eC
	T5	23.52 aA	21.25 aA	18.37 aA	16.36 aA	51.44 aA	31.58 aA	8.26 eC	12.25 fC
10–20	CK	8.56 fB	6.85 fB	5.47 fB	4.46 fB	41.21 cB	18.92 eB	17.59 aB	22.22 aB
	T1	11.52 eB	10.37 eB	9.12 eB	5.51 eB	41.46 cB	23.23 dB	17.50 aB	17.94 bB
	T2	13.54 dB	10.54 dB	10.48 dB	6.99 dB	44.63 bA	23.29 dB	16.60 bB	17.94 bB
	T3	15.56 cB	12.69 cB	12.49 cB	8.98 cB	45.47 bB	25.09 cB	14.78 cB	17.56 bB
	T4	16.53 bB	14.66 bB	13.36 bB	10.10 bB	48.24 aB	28.57 bB	11.89 dB	15.98 cB
	T5	17.79 aB	16.46 aB	14.89 aB	11.91 aB	49.33 aB	30.52 aB	10.82 eB	14.20 dB
20–30	CK	5.47 fC	4.43 fC	4.15 fC	2.32 fC	34.73 eC	14.84 fC	24.55 aA	21.03 bC
	T1	7.26 eC	6.21 eC	5.84 eC	4.10 eC	33.10 fC	16.77 eC	20.88 bA	22.49 aA
	T2	7.72 dC	6.65 dC	6.54 dC	4.85 dC	36.30 dB	19.82 dC	20.67 bA	22.80 aA
	T3	8.79 cC	7.67 cC	7.59 cC	5.84 cC	38.36 cC	22.98 cC	18.15 cA	21.39 bA
	T4	11.68 bC	9.65 bC	8.44 bC	7.39 bC	42.43 bC	25.08 bC	15.22 dA	19.00 cA
	T5	12.56 aC	11.36 aC	9.45 aC	8.44 aC	46.50 aC	29.88 aB	14.12 eA	18.24 dA

Significant differences across treatments within the same soil layer are denoted by different lowercase letters (*p* < 0.05), and significant differences across soil layers under the same treatment are denoted by distinct uppercase letters (*p* < 0.05). The same applies hereinafter.

**Table 5:** Contents of ROC in soil aggregates of varying size classes and their contribution rates to total organic carbon under different biochar application rates.

Soil Depth (cm)	Treatment	ROC Content (g/kg)				ROC Contribution Rates (%)			
		>2 mm	2–0.25 mm	0.25–0.053 mm	<0.053 mm	>2 mm	2–0.25 mm	0.25–0.053 mm	<0.053 mm
0–10	CK	3.12 fA	2.46 fA	1.54 fA	1.90 fA	13.23 dA	6.30 eA	3.15 bB	5.93 aA
	T1	5.24 eA	4.16 eA	3.31 eA	2.25 eA	15.77 cA	8.22 dA	4.04 aA	4.16 dA
	T2	5.77 dA	5.03 dA	4.24 dA	3.35 dA	15.47 cA	8.89 cA	3.94 aA	5.18 bA
	T3	6.63 cA	5.75 cA	4.76 cA	4.05 cA	16.51 bA	9.86 bA	3.18 bA	5.00 cA
	T4	7.52 bA	6.71 bA	5.58 bA	5.22 bA	16.60 bA	10.07 bA	2.87 cA	5.00 cA
	T5	8.51 aA	7.21 aA	6.23 aA	5.48 aA	18.60 aA	10.71 aA	2.80 cA	4.10 dA
10–20	CK	2.08 fB	1.35 fB	1.08 fB	0.64 fB	10.02 cdB	3.73 fB	3.49 aA	3.19 bB
	T1	2.42 eB	1.83 eB	1.41 eB	0.95 eB	8.70 eB	4.11 eB	2.71 cB	3.11 bcB
	T2	2.91 dB	2.40 dB	1.92 dB	1.38 dB	9.60 dB	5.30 dB	3.04 bB	3.55 aB
	T3	3.51 cB	2.95 cB	2.40 cB	1.51 cB	10.25 cB	5.83 cB	2.83 bcB	2.95 cC
	T4	4.58 bB	3.54 bB	2.76 bB	2.15 bB	13.36 bB	6.91 bB	2.46 dB	3.40 aB
	T5	6.21 aB	4.56 aB	3.32 aB	2.53 aB	17.21 aB	8.46 aB	2.41 dB	3.02 bcB
20–30	CK	0.74 eC	0.64 fC	0.46 eC	0.34 fC	4.72 cC	2.16 dC	2.72 bC	3.09 bB
	T1	0.95 dC	0.84 eC	0.64 dC	0.47 eC	4.34 cC	2.26 dC	2.29 cC	2.56 dC
	T2	1.35 cC	1.06 dC	0.85 cC	0.67 dC	6.34 bC	3.15 cC	2.69 bC	3.15 bC
	T3	1.73 bC	1.47 cC	1.21 bC	0.96 cC	7.54 aC	4.41 bC	2.89 aB	3.51 aB
	T4	1.83 abC	1.59 bC	1.29 abC	1.08 bC	6.66 bC	4.14 bC	2.33 cB	2.77 cC
	T5	1.95 aC	1.79 aC	1.52 aC	1.34 aC	7.21 aC	4.70 aC	2.28 cB	2.90 cB

**Table 6:** Contents of EOC in soil aggregates of varying size and their contribution proportions to total organic carbon under different biochar application rates.

Soil Depth (cm)	Treatment	EOC Content (g/kg)				EOC Contribution Rates (%)			
		>2 mm	2–0.25 mm	0.25–0.053 mm	<0.053 mm	>2 mm	2–0.25 mm	0.25–0.053 mm	<0.053 mm
0–10	CK	3.15 fA	2.51 eA	1.92 fA	1.48 fB	13.38 dA	6.44 cA	3.92 aC	4.61 aC
	T1	3.64 eA	3.40 dA	2.64 eA	2.40 eA	10.96 eB	6.71 cA	3.22 bC	4.45 abB
	T2	5.05 dA	4.42 cA	3.24 dA	2.75 dA	13.54 dA	7.81 bA	3.01 cC	4.25 bC
	T3	6.27 cA	5.49 bA	4.41 cA	3.39 cA	15.60 bA	9.41 aA	2.95 cdC	4.18 bC
	T4	6.83 bA	6.46 aA	5.43 bA	4.42 bA	15.07 cB	9.69 aA	2.79 dB	4.23 bB
	T5	7.52 aA	6.56 aA	5.79 aA	4.76 aA	16.44 aC	9.75 aA	2.60 eB	3.57 cB
10–20	CK	2.47 fB	2.25 fB	1.50 fB	1.69 eA	11.88 eB	6.20 dA	4.83 aB	8.41 aA
	T1	3.47 eB	2.58 eB	2.35 eB	2.07 dB	12.50 dA	5.79 eB	4.50 bB	6.74 bA
	T2	3.93 dB	2.94 dB	2.48 dB	2.20 cB	12.95 cdB	6.49 dB	3.93 cB	5.65 cB
	T3	4.56 cB	3.53 cB	2.88 cB	2.49 bB	13.32 cB	6.98 cC	3.41 dB	4.87 dB
	T4	5.54 bB	4.56 bB	3.33 bB	2.84 aB	16.18 bA	8.88 bB	2.97 eB	4.49 eB
	T5	6.45 aB	5.32 aB	3.55 aB	2.92 aB	17.89 aA	9.86 aA	2.58 fB	3.49 fB
20–30	CK	1.67 fC	1.40 fC	1.08 fC	0.70 fC	10.60 eC	4.69 eB	6.39 cA	6.36 cB
	T1	2.08 eC	1.63 eC	1.47 eC	1.24 eC	9.48 fC	4.41 eC	5.26 bA	6.79 bcA
	T2	2.51 dC	1.95 dC	1.65 dC	1.49 dC	11.79 dC	5.81 dC	5.22 bA	6.99 bA
	T3	3.53 cC	2.79 cC	2.18 cC	2.40 cB	15.41 cA	8.37 cB	5.22 bA	8.80 aA
	T4	4.51 bC	3.52 bC	3.22 bC	2.55 bC	16.40 bA	9.14 bB	5.82 aA	6.56 cA
	T5	4.70 aC	3.68 aC	3.46 aB	2.80 aC	17.39 aB	9.67 aA	5.18 bA	6.05 dA

**Table 7:** Contents of POC in soil aggregates of varying size classes and their contribution rates to total OC under different biochar amendment levels.

Soil Depth (cm)	Treatment	POC Content (g/kg)				POC Contribution Rates (%)			
		>2 mm	2–0.25 mm	0.25–0.053 mm	<0.053 mm	>2 mm	2–0.25 mm	0.25–0.053 mm	<0.053 mm
0–10	CK	2.35 fA	2.06 fA	1.64 fA	1.23 fA	9.98 fB	5.29 fA	3.34 aC	3.83 dB
	T1	3.79 eA	3.20 eA	2.56 eA	1.77 eA	11.39 eA	6.33 eA	3.12 bC	3.28 eB
	T2	4.93 dA	4.39 dA	3.59 dA	2.88 dA	13.22 dA	7.76 dA	3.33 aC	4.45 aB
	T3	6.16 cA	5.35 cA	4.28 cA	3.37 cA	15.33 cA	9.17 cA	2.86 cB	4.16 bcB
	T4	7.55 bA	6.46 bA	5.31 bA	4.43 bA	16.68 bA	9.69 bA	2.73 cC	4.24 abB
	T5	8.19 aA	7.22 aA	6.23 aA	5.27 aA	17.92 aA	10.73 aA	2.80 cB	3.95 cdA
10–20	CK	2.13 fB	1.51 fB	1.24 eB	0.95 eB	10.27 eB	4.16 eC	4.00 aB	4.71 abA
	T1	2.52 eB	2.26 eB	1.87 dB	1.29 dB	9.08 fB	5.07 dB	3.58 bB	4.19 cA
	T2	3.55 dB	2.51 dB	2.34 cB	1.78 cB	11.71 cB	5.55 cB	3.71 bB	4.57 bB
	T3	3.66 cB	2.64 cB	2.45 cB	1.88 cB	10.71 dB	5.22 dC	2.90 dB	3.67 dC
	T4	4.85 aB	3.85 aB	3.63 aB	3.07 bB	14.14 aB	7.51 aB	3.23 cB	4.87 aA
	T5	4.47 bB	3.46 bB	2.61 bB	3.24 aB	12.39 bB	6.41 bB	1.89 eC	3.86 dA
20–30	CK	1.72 fC	1.35 fC	1.03 fC	0.51 eC	10.89 aA	4.53 dB	6.12 aA	4.66 bA
	T1	1.92 eC	1.50 eC	1.18 eC	0.75 dC	8.73 cB	4.05 eC	4.22 cdA	4.12 cA
	T2	2.31 dC	1.81 dC	1.50 dC	1.08 cC	10.84 aC	5.39 cB	4.73 bA	5.07 aA
	T3	2.52 cC	2.12 cC	1.79 cC	1.40 aC	11.00 aB	6.36 abB	4.28 cA	5.12 aA
	T4	3.03 aC	2.53 aC	2.26 aC	1.73 aC	11.01 aC	6.58 aC	4.08 dA	4.45 bB
	T5	2.81 bC	2.33 bC	2.11 bC	1.53 bC	10.39 bC	6.12 bB	3.15 eA	3.30 dB

**Table 8:** Contents of MBC in soil aggregates of varying size classes and their contribution rates to total organic carbon under different biochar application rates.

Soil Depth (cm)	Treatment	MBC Content (mg/kg)				MBC Contribution Rates (%)			
		>2 mm	2–0.25 mm	0.25–0.053 mm	<0.053 mm	>2 mm	2–0.25 mm	0.25–0.053 mm	<0.053 mm
0–10	CK	142.89 fA	161.27 fA	122.22 fA	98.34 fA	0.61 dA	0.41 aA	0.25 aC	0.31 aC
	T1	188.82 eA	166.72 eA	140.53 eA	113.87 eA	0.57 eA	0.33 dA	0.17 bB	0.21 bC
	T2	226.89 dA	211.33 dA	174.47 dA	130.62 dA	0.61 dA	0.37 cA	0.16 cC	0.20 bcC
	T3	269.24 cA	242.98 cA	205.96 cA	160.60 cA	0.67 bA	0.42 aA	0.14 dC	0.20 cC
	T4	323.52 aA	280.59 aA	236.45 aA	191.97 aA	0.71 aA	0.42 aA	0.12 eC	0.18 dC
	T5	289.24 bA	262.98 bA	222.90 bA	174.03 bA	0.63 cA	0.39 bA	0.10 fC	0.13 eC
10–20	CK	127.51 fB	113.80 fB	103.68 fB	86.08 fB	0.61 bA	0.31 dB	0.33 aA	0.43 aA
	T1	137.62 eB	123.70 eB	111.19 eB	94.91 eB	0.50 dB	0.28 eB	0.21 cA	0.31 cA
	T2	169.38 dB	154.86 dB	143.35 dB	126.61 dB	0.56 cB	0.34 cB	0.23 bB	0.33 bB
	T3	203.34 cB	188.92 cB	176.87 cB	154.73 cB	0.59 bC	0.37 bB	0.21 cB	0.30 cdB
	T4	230.81 aB	215.66 aB	203.94 aB	188.52 aB	0.67 aB	0.42 aAB	0.18 dB	0.30 dB
	T5	218.85 bB	203.81 bB	191.84 bB	175.51 bA	0.61 bB	0.38 bB	0.14 eB	0.21 eB
20–30	CK	79.07 fC	66.09 fC	50.16 fC	37.82 fC	0.50 dB	0.22 dC	0.30 aB	0.34 cB
	T1	88.21 eC	74.71 eC	57.21 eC	45.24 eC	0.40 eC	0.20 eC	0.21 dA	0.25 eB
	T2	118.64 dC	107.40 dC	87.65 dC	76.44 dC	0.56 cB	0.32 cC	0.28 bA	0.36 bA
	T3	150.00 cC	136.07 cC	116.34 cC	104.85 cC	0.66 aB	0.41 aA	0.28 bA	0.38 aA
	T4	170.07 aC	157.93 aC	137.33 aC	125.72 aC	0.62 bC	0.41 aB	0.25 cA	0.32 dA
	T5	163.52 bC	148.96 bC	129.01 bC	116.35 bB	0.61 bB	0.39 bA	0.19 eA	0.25 eA

**Table 9:** Contents of DOC in soil aggregates of varying size and their contribution proportions to total organic carbon under different biochar application rates.

Soil Depth (cm)	Treatment	DOC Content (mg/kg)				DOC Contribution Rates (%)			
		>2 mm	2–0.25 mm	0.25–0.053 mm	<0.053 mm	>2 mm	2–0.25 mm	0.25–0.053 mm	<0.053 mm
0–10	CK	22.64 fA	20.29 fA	18.41 fA	15.33 fA	0.10 aA	0.05 aA	0.04 aA	0.05 bA
	T1	25.57 eA	22.47 eA	19.49 eA	16.90 eA	0.08 cA	0.04 bA	0.02 bB	0.03 bB
	T2	31.63 dA	27.55 dA	24.54 dA	21.27 dA	0.09 bA	0.05 aA	0.02 bB	0.03 bA
	T3	33.53 cA	28.75 cA	25.60 cA	22.67 cA	0.08 cA	0.05 aA	0.02 bA	0.03 bA
	T4	38.75 aA	33.66 aA	30.55 aA	27.60 aA	0.09 bA	0.05 aA	0.02 bB	0.03 bA
	T5	36.52 bA	31.64 bA	27.55 bA	24.94 bA	0.08 cA	0.05 aA	0.01 cB	0.02 cA
10–20	CK	13.86 fB	11.55 fB	9.33 fB	10.34 fB	0.07 aB	0.03 bB	0.03 aB	0.05 aA
	T1	17.73 eB	16.66 eB	15.50 eB	11.66 eB	0.06 bB	0.04 aA	0.03 aA	0.04 bA
	T2	19.53 dB	18.32 dB	16.79 dB	13.76 dB	0.06 bB	0.04 aB	0.03 aA	0.03 cA
	T3	20.68 cB	19.42 cB	18.49 cB	15.83 cB	0.06 bB	0.04 aB	0.02 bA	0.03 cA
	T4	23.69 aB	22.27 aB	20.35 aB	18.36 aB	0.07 aB	0.04 aB	0.02 bB	0.03 cA
	T5	22.11 bB	20.31 bB	19.49 bB	17.33 bB	0.06 bB	0.04 aB	0.01 cB	0.02 dA
20–30	CK	7.42 fC	6.18 fC	4.87 fC	3.08 fC	0.05 cC	0.02 cC	0.03 aB	0.03 aB
	T1	10.32 eC	8.97 eC	7.41 eC	5.35 eC	0.05 cC	0.02 cB	0.03 aA	0.03 aB
	T2	11.18 dC	9.51 dC	8.06 dC	5.97 dC	0.05 cC	0.03 bC	0.03 aA	0.03 aA
	T3	13.60 cC	11.65 cC	10.25 cC	8.44 cC	0.06 bB	0.03 bC	0.02 bA	0.03 aA
	T4	18.21 aC	16.44 aC	14.76 aC	12.42 aC	0.07 aB	0.04 aB	0.03 aA	0.03 aA
	T5	14.31 bC	12.36 bC	10.92 bC	8.87 bC	0.05 cC	0.03 bC	0.02 bA	0.02 bA

**Table 10:** Contents of black carbon (BC) in soil aggregates of varied size fractions and their contribution proportions to total organic carbon under different biochar application rates.

Soil Depth (cm)	Treatment	BC Content (g/kg)				BC Contribution Rates (%)			
		>2 mm	2–0.25 mm	0.25–0.053 mm	<0.053 mm	>2 mm	2–0.25 mm	0.25–0.053 mm	<0.053 mm
0–10	CK	3.48 fA	2.85 fA	1.90 fA	2.34 fA	14.79 eA	7.29 fA	3.87 cC	7.32 aA
	T1	5.02 eA	4.36 eA	3.84 eA	3.32 eA	15.11 eA	8.61 eA	4.69 aC	6.15 bcC
	T2	6.31 dA	5.34 dA	4.50 dA	4.11 dA	16.91 dA	9.45 dB	4.18 bC	6.35 bC
	T3	7.75 cA	6.83 cA	5.66 cA	5.02 cA	19.30 cA	11.72 cA	3.78 cC	6.20 bcC
	T4	9.55 bA	8.66 bA	6.80 bA	6.20 bA	21.08 bA	13.00 bB	3.50 dC	5.93 cB
	T5	11.15 aA	10.47 aA	8.89 aA	8.01 aA	24.39 aA	15.56 aA	4.00 bcC	6.00 cC
10–20	CK	2.75 fB	2.33 fB	1.58 fB	1.31 fB	13.23 fB	6.44 fB	5.10 cB	6.54 cB
	T1	3.96 eB	3.67 eB	2.76 eB	2.06 eB	14.27 eB	8.23 eA	5.30 bcB	6.70 cB
	T2	5.21 dB	4.62 dB	3.46 dB	3.11 dB	17.18 dA	10.21 dA	5.47 bB	7.98 aB
	T3	6.24 cB	5.92 cB	4.89 cB	4.19 cB	18.25 cB	11.71 cA	5.79 aB	8.19 aB
	T4	7.05 bB	6.94 bB	5.35 bB	4.88 bB	20.57 bB	13.53 bA	4.76 dB	7.72 bA
	T5	8.18 aB	7.97 aB	6.34 aB	5.60 aB	22.67 aB	14.77 aB	4.61 dB	6.68 cB
20–30	CK	1.73 fC	1.43 fC	1.19 fC	0.82 fC	10.95 fC	4.78 fC	7.03 aA	7.47 dA
	T1	2.58 eC	2.26 eC	1.78 eC	1.50 eC	11.77 eC	6.10 eB	6.38 cA	8.23 bA
	T2	3.09 dC	2.70 dC	2.10 dC	1.89 dC	14.54 dB	8.03 dC	6.65 bA	8.89 aA
	T3	3.96 cC	3.29 cC	2.62 cC	2.38 cC	17.30 cC	9.85 cB	6.26 cA	8.70 aA
	T4	5.12 bC	4.42 bC	3.34 bC	3.10 bC	18.59 bC	11.49 bC	6.03 dA	7.96 cA
	T5	5.85 aC	5.43 aC	3.99 aC	3.62 aC	21.66 aC	14.29 aC	5.96 dA	7.83 cA

## Data Availability

The original contributions presented in this study are included in the article. Further inquiries can be directed to the corresponding authors.
